# Tuning the Fluorescence and the Intramolecular Charge Transfer of Phenothiazine Dipolar and Quadrupolar Derivatives by Oxygen Functionalization

**DOI:** 10.1021/jacs.1c04173

**Published:** 2021-06-23

**Authors:** Yogajivan Rout, Chiara Montanari, Erika Pasciucco, Rajneesh Misra, Benedetta Carlotti

**Affiliations:** † Department of Chemistry, Indian Institute of Technology, Indore 453552, India; ‡ Department of Chemistry, Biology and Biotechnology, University of Perugia, via elce di sotto 8, 06123 Perugia, Italy

## Abstract

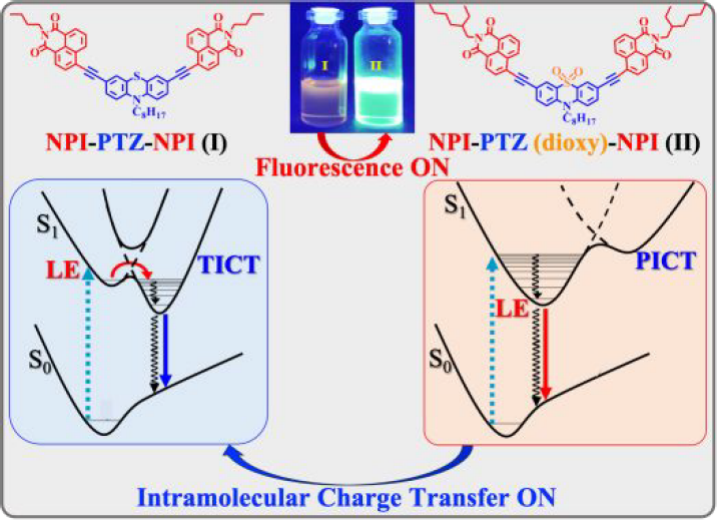

A series of new naphthalimide and phenothiazine-based push–pull systems (**NPI-PTZ1–5**), in which we structurally modulate the oxidation state of the sulfur atom in the thiazine ring, i.e., S(II), S(IV), and S(VI), was designed and synthesized by the Pd-catalyzed Sonogashira cross-coupling reaction. The effect of the sulfur oxidation state on the spectral, photophysical, and electrochemical properties was investigated. The steady-state absorption and emission results show that oxygen functionalization greatly improves the optical (absorption coefficient and fluorescence efficiency) and nonlinear optical (hyperpolarizability) features. The cyclic voltammetry experiments and the quantum mechanical calculations suggest that phenothiazine is a stronger electron donor unit relative to phenothiazine-5-oxide and phenothiazine-5,5-dioxide, while the naphthalimide is a strong electron acceptor in all cases. The advanced ultrafast spectroscopic measurements, transient absorption, and broadband fluorescence up conversion give insight into the mechanism of photoinduced intramolecular charge transfer. A planar intramolecular charge transfer (PICT) and highly fluorescent excited state are populated for the oxygen-functionalized molecules **NPI-PTZ2,3** and **NPI-PTZ5**; on the other hand, a twisted intramolecular charge transfer (TICT) state is produced upon photoexcitation of the oxygen-free derivatives **NPI-PTZ1** and **NPI-PTZ4**, with the fluorescence being thus significantly quenched. These results prove oxygen functionalization as a new effective synthetic strategy to tailor the photophysics of phenothiazine-based organic materials for different optoelectronic applications. While oxygen-functionalized compounds are highly fluorescent and promising active materials for current-to-light conversion in organic light-emitting diode devices, oxygen-free systems show very efficient photoinduced ICT and may be employed for light-to-current conversion in organic photovoltaics.

## Introduction

The design and synthesis of new push–pull organic materials has emerged as a hot area of research over the past two decades because of their potential application in organic light-emitting diodes (OLEDs), nonlinear optics, photovoltaic cells and bioimaging.^1−4^ These push–pull semiconducting materials show unique electronic and photonic features which may be tuned and improved by easy synthetic modifications. In these push–pull chromophores, heterocyclic derivatives (which contain nitrogen, oxygen, and sulfur) were mainly introduced in the π-conjugated systems to modulate their photophysical and electrochemical properties.^5−7^ Many studies report on the preparation of new organic push–pull materials and their good device or biological performance. However, a deep understanding of their successful application by studying their detailed excited-state dynamics and mechanism is rarely reached. This could be extremely valuable in order to get feedback and guidance about new optimal design and synthetic strategies.

Perylenediimide (PDI),^8^ naphthalenediimide (NDI),^9^ and diketopyrrolopyrrole (DPP)^7^ have been largely used as electron-acceptor units in new materials for optoelectronic and biochemical technologies because of their excellent chemical, thermal, and photostability. However, compared to the PDI, NDI, and DPP analogues, 1,8-naphthalimide (NPI) derivatives show the same positive properties while being even more promising dyes,^10−19^ because they are less affected by aggregation issues. More recently, phenothiazine (PTZ) was often used as the active component in push–pull chromophores because of its strong electron-donating capability.^20^ The PTZ unit is an electron-rich tricyclic heteroarene with nonplanar butterfly structure, characterized by the presence of powerful electron-donor sulfur and nitrogen atoms.^21^ In the literature, the photophysical properties and the HOMO–LUMO energy levels of PTZ derivatives have been easily modulated by substitutions at the nitrogen and the 3,7-positions of the phenothiazine unit.^22,23^ Our group has functionalized the 3,7-positions of the phenothiazine unit by using polycyclic aromatic hydrocarbons of increasing complexity^24^ or strong acceptors such as benzothiadiazole^25^ or tetracyanobutadiene.^26,27^ In the literature, push–pull phenothiazine–naphthalimide systems have been successfully employed in some optoelectronic applications.^28−38^ However, the research on varying the oxidation state of the sulfur atom (sulfides, sulfoxides, and sulfones) in the thiazine ring of phenothiazine is still very limited.^39−41^ In this study, we have synthesized new naphthalimide and phenothiazine-based systems, in which we have changed the oxidation state of the sulfur atom on the thiazine ring to investigate its effect on the photonic properties of the obtained materials.

**Chart 1:**
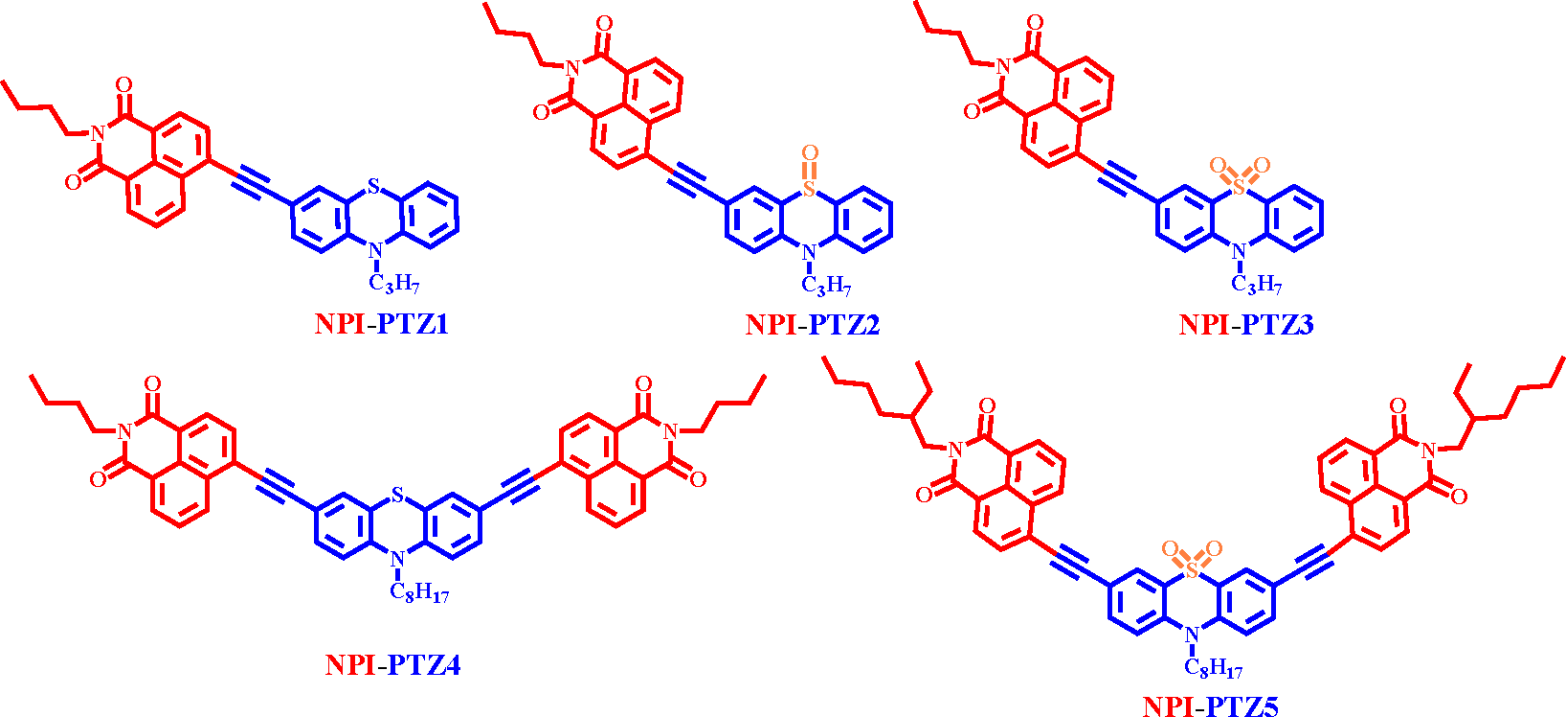
Molecular Structures of the Investigated Compounds

In particular, herein we report the synthesis of five phenothiazine and naphthalimide-based compounds, with both dipolar (D−π–*A*) and quadrupolar (A−π–D−π–*A*) structures, shown in Chart 1. In these phenothiazine derivatives, we alter the oxidation state (i.e., S(II), S(IV), and S(VI)) of the sulfur atom. In the push–pull chromophores **NPI-PTZ1** and **NPI-PTZ4**, phenothiazine was used as the donor, whereas in **NPI-PTZ2**, **NPI-PTZ3**, and **NPI-PTZ5**, phenothiazine 5-oxide and phenothiazine 5,5-dioxide were used as donor units. With this study, we investigate the effect of the phenothiazine oxygen functionalization on the spectral, photophysical, and electrochemical features of these molecules. To reach this goal, we employ not only cyclic voltammetry and steady-state spectroscopy but also advanced time-resolved spectroscopic techniques, such as nanosecond and femtosecond transient absorption as well as broadband fluorescence up conversion to gain a deep understanding of the excited-state behavior.

## Results and Discussion

### Synthesis and Characterization

**Scheme 1:**
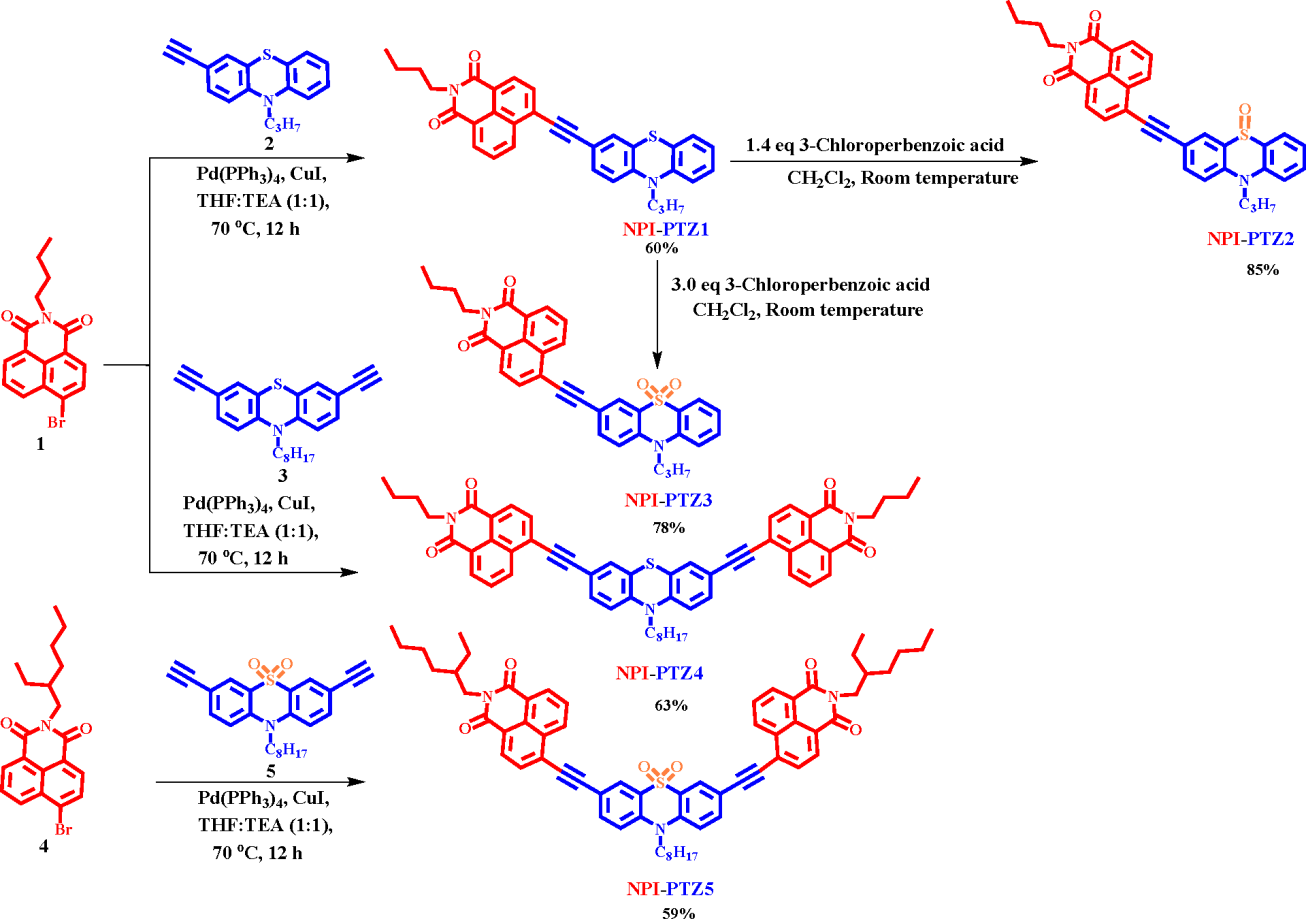
Synthesis of the Compounds under Investigation

The detailed synthetic routes for 1,8-naphthalimide-functionalized phenothiazine-based chromophores are shown in Scheme 1. The 1,8-naphthalimide substituted **NPI-PTZ1** chromophore was synthesized by the Sonogashira cross coupling reaction of 3-ethynyl-10-propyl-10H-phenothiazine **2** with one equivalent of 6-bromo-2-butyl-1H benzo[de]isoquinoline-1,3(2H)-dione **1** in the presence of Pd(PPh_3_)_4_ as the catalyst in 60% yield. The reaction of **NPI-PTZ1** with 1.4 equiv of 3-chloroperbenzoic acid in dichloromethane solution at room temperature for 1 h resulted in **NPI-PTZ2** with 85% yield, whereas the dioxide derivative **NPI-PTZ3** was synthesized in 78% yield by using three equivalents of 3-chloroperbenzoic acid in the same conditions. The push–pull chromophores **NPI-PTZ4** and **NPI-PTZ5** were synthesized by the Sonogashira cross coupling of 3,7-diethynyl-10-octyl-10H-phenothiazine **3** with two equivalents of 6-bromo-2-butyl-1H benzo[de]isoquinoline-1,3(2H)-dione **1**, and of 3,7-diethynyl-10-octyl-10H-phenothiazine 5,5-dioxide **5**(42) with two equivalents of 6-bromo-2-(2-ethylhexyl)-1H-benzo[de]isoquinoline-1,3(2H)-dione **4**, in 63% and 58% yields, respectively. The chemical structures of the synthesized molecules were confirmed by ^1^H and ^13^C NMR, HRMS, and MALDI-TOF mass spectrometry techniques, and the products are readily soluble in common organic solvents (see the Supporting Information).

### Spectral and Fluorescence Properties

**Figure 1:**
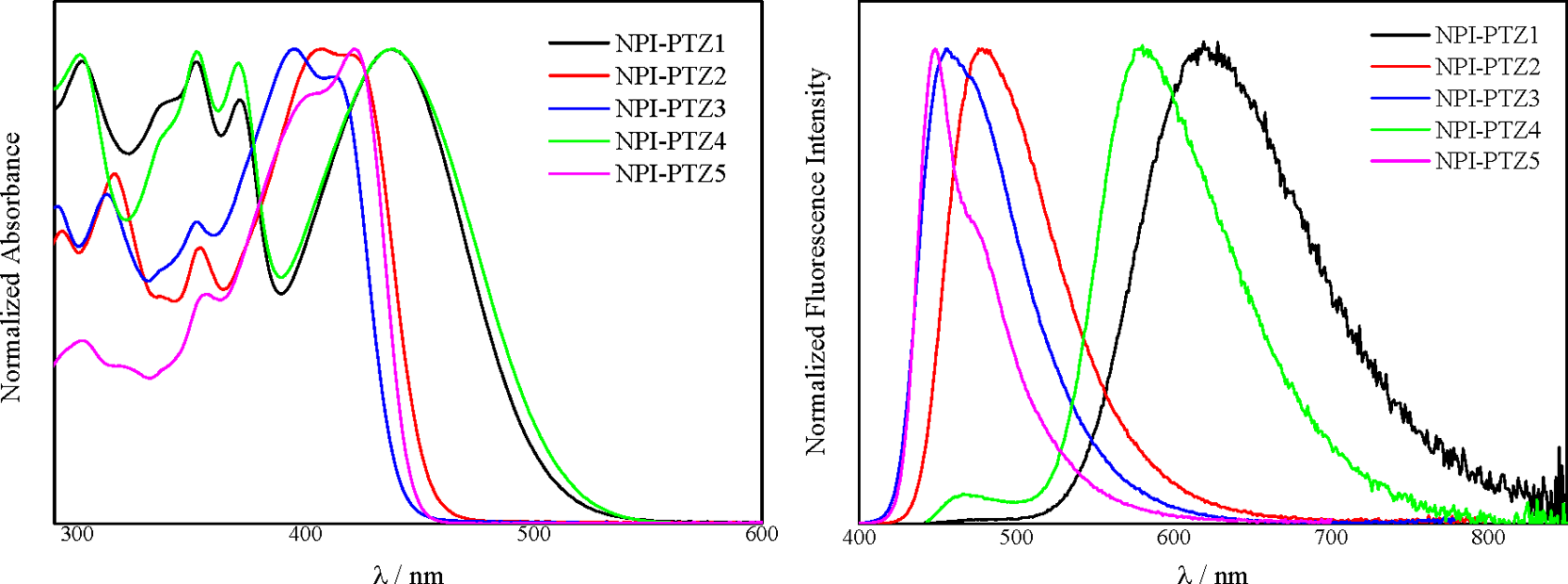
Normalized absorption (left) and emission (right) spectra of the investigated compounds in Tol.

Figure 1 shows the absorption and emission spectra of the investigated compounds in toluene. The lower energetic absorption band and the emission spectrum appear structured for the oxygen-functionalized compounds **NPI-PTZ2**, **NPI-PTZ3**, and **NPI-PTZ5** but broader and structureless for the oxygen-free **NPI-PTZ1** and **NPI-PTZ4**. In all cases, the oxygen functionalization of the sulfur atom of the phenothiazine implies a blue shift of the absorption and emission spectra. For instance, the absorption maximum is at 437 nm for **NPI-PTZ1**, 407 nm for **NPI-PTZ2**, and 395 nm for **NPI-PTZ3**. An analogous trend was also observed when considering the emission maxima or the two branched systems (see Table 1). The bathochromically shifted spectra observed for the oxygen-free relative to the oxygen-functionalized derivatives may indicate that phenothiazine is a stronger donor unit relative to phenothiazine 5-oxide and phenothiazine 5,5-dioxide.^41^

**Table 1:** Spectral (Absorption and Emission Maxima, Molar Absorption Coefficient, Optical Band Gap, and Stokes Shift) and Fluorescence (Quantum Yield (ϕ_F_), Lifetime (τ_F_), and Rate Constant (*k*_F_)) Properties of the Investigated Compounds in Tol^a^

compound	λ_Abs_ (nm)	ε (M^–1^ cm^–1^)	*E*_g,opt_ (eV)	λ_Em_ (nm)	Δν (cm^–1^)	ϕ_F_	τ_F_ (ns)	*k*_F_ (s^–1^)
**NPI-PTZ1**	437	17200	2.28	619	6730	0.51	5.17	9.7 × 10^7^
**NPI-PTZ2**	407	23200	2.63	478	3650	0.87	2.22	3.9 × 10^8^
**NPI-PTZ3**	395	27100	2.71	456	3390	0.71	1.69	4.1 × 10^8^
**NPI-PTZ4**	438	23100	2.28	580	5590	0.66	3.50	1.9 × 10^8^
**NPI-PTZ5**	421	64900	2.72	448	1430	0.75	1.24	6.0 × 10^8^

a The radiative rate constants were obtained from the experimentally observed fluorescence quantum yields and lifetimes as *k*_F_ = ϕ_F_/τ_F_.

The optical band gap (*E*_g,opt_), estimated from the onset wavelength of the absorption spectrum, increases upon increasing the number of oxygen atoms attached to the phenothiazine sulfur (2.28, 2.63, and 2.71 eV for **NPI-PTZ1**, **NPI-PTZ2**, and **NPI-PTZ3**, respectively) while being unaffected by the dipolar versus quadrupolar structure (2.28 and 2.72 eV for **NPI-PTZ4** and **NPI-PTZ5**, respectively). The Stokes shift values are quite large for the oxygen-free **NPI-PTZ1** and **NPI-PTZ4** molecules (6730 and 5590 cm^–1^, respectively) and are significantly reduced in the phenothiazine oxide and dioxide derivatives (3650, 3390, and 1430 cm^–1^ for **NPI-PTZ2**, **NPI-PTZ3**, and **NPI-PTZ5**, respectively). These results suggest a more rigid molecular structure for the oxygen-functionalized relative to the oxygen-free compounds. The molar absorption coefficients (ε in Table 1 and Figure S19) are found to increase upon increasing the number of oxygen atoms linked to the phenothiazine (17200, 23200, and 27100 M^–1^cm^–1^ for **NPI-PTZ1**, **NPI-PTZ2**, and **NPI-PTZ3**, respectively) and upon passing from the monobranched to the two-branched systems (23100 and 64900 M^–1^cm^–1^ for **NPI-PTZ4** and **NPI-PTZ5**, respectively).

The fluorescence quantum yields are significant (51–87%) in toluene (Table 1). The obtained values are generally higher for the oxygen-functionalized than for the oxygen-free derivatives and enhanced in the quadrupolar systems relative to the dipolar analogues. Fluorescence lifetimes of several nanoseconds were measured through time correlated single-photon counting measurements. The lifetime values are shorter in the phenothiazine oxide and dioxide compared to the phenothiazine derivatives (5.17, 2.22, and 1.69 ns for **NPI-PTZ1**, **NPI-PTZ2**, and **NPI-PTZ3**, respectively) and in the two-branched relative to the monobranched molecules (3.50 and 1.24 ns for **NPI-PTZ4** and **NPI-PTZ5**, respectively). As a result, the radiative rate constants (*k*_F_ = ϕ_F_/τ_F_) are enhanced in the oxygen-functionalized derivatives and in the quadrupolar systems (see Table 1). Our findings clearly demonstrate the positive effect of the oxygen functionalization on the light absorption and emission capability of these phenothiazine–naphthalimide systems.

### Electrochemical Properties

Cyclic voltammetry was used to explore the redox behavior and potentials of the investigated samples. The electrochemical properties are depicted in Figure S16. The electrochemical data of all the derivatives are collected in Table S1. The mono and di-1,8-naphthalimide-based phenothiazine derivative **NPI-PTZ1** and **NPI-PTZ4** exhibit a single reversible reduction wave at −1.23 V corresponding to the 1,8-naphthalimide acceptor unit. Similarly, **NPI-PTZ2**, **NPI-PTZ3**, and **NPI-PTZ5** show a reversible reduction wave at −1.20, −1.21, and −1.23 V corresponding to the reduction of the same unit. The oxygen-free compounds, **NPI-PTZ1** and **NPI-PTZ4**, exhibit a single reversible oxidation wave at 0.78 and 0.83 V attributed to the phenothiazine strong donor. In contrast, **NPI-PTZ2**, **NPI-PTZ3**, and **NPI-PTZ5** exhibit a single irreversible oxidation wave at 1.41, 1.50, and 1.56 V corresponding to the phenothiazine 5-oxide and phenothiazine 5,5-dioxide units. Therefore, on the anodic side, the oxidation waves of the oxygen-functionalized phenothiazine derivatives are shifted toward more positive values compared to **NPI-PTZ1** and **NPI-PTZ4** because of the increase of the sulfur oxidation state on the thiazine ring. The HOMO and LUMO energy levels of **NPI-PTZ1**, **NPI-PTZ4**, **NPI-PTZ2**, **NPI-PTZ3**, and **NPI-PTZ5** were estimated by using the first onset potentials of oxidation and reduction waves at −5.05, −5.12, −5.72, −5.84, and −5.85 eV and −3.30, −3.26, −3.29, −3.22, and −3.25 eV, respectively. These results indicate that increasing of sulfur oxidation state has more effect on the HOMO compared to the LUMO energy levels.

### Theoretical Calculations

**Figure 2:**
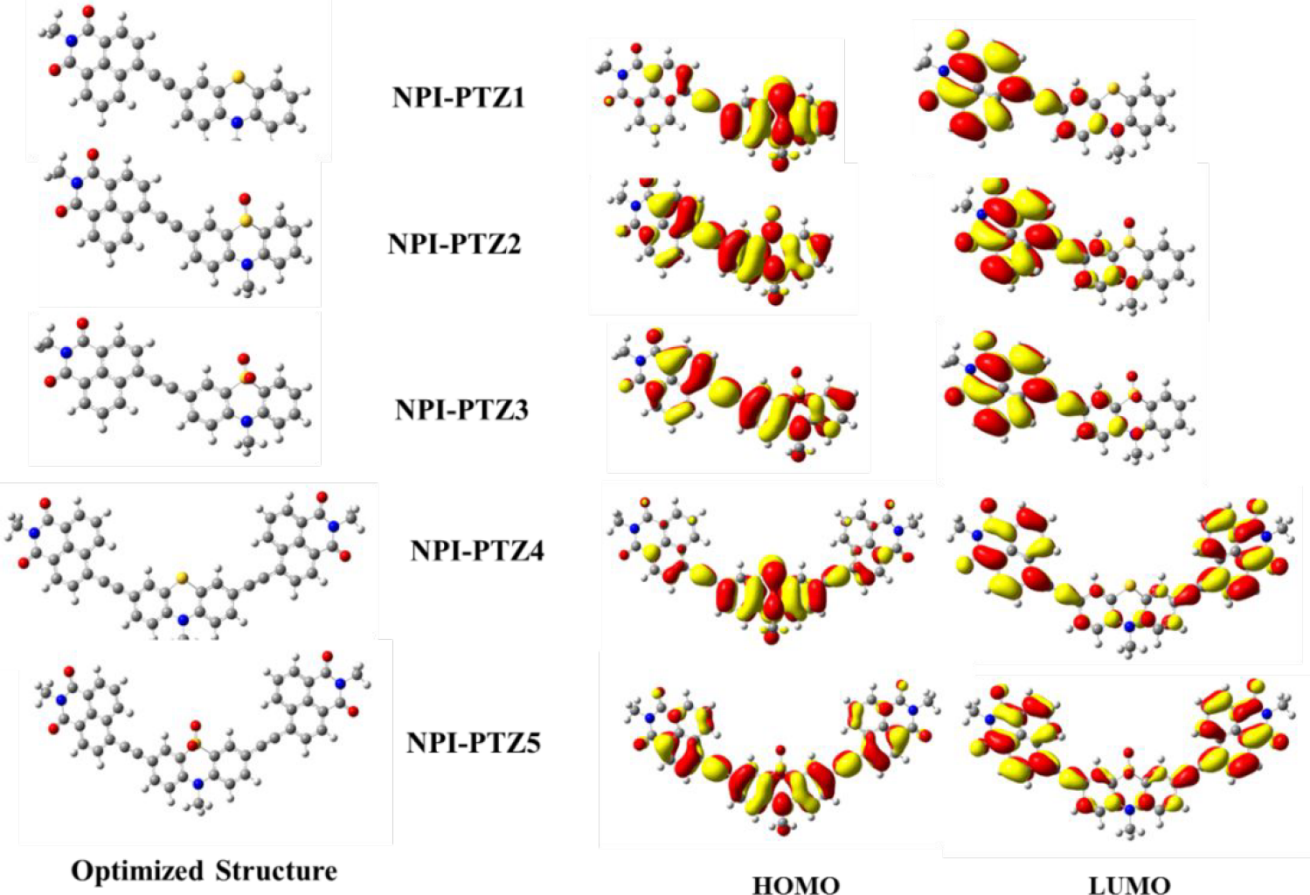
Optimized ground-state geometry and frontier HOMO and LUMO orbitals obtained by DFT calculations (B3LYP functional/6-31G** basis set).

The structural and electronic properties of the NPI-PTZ molecules (containing methyl substituents instead of the alkyl ones) were investigated by DFT calculations.^43^ The simpler methyl substituents were used in order to save computational time. Molecular geometries and frontier molecular orbitals of all the investigated compounds are shown in Figure 2. They exhibit nonplanar structures due to the presence of phenothiazine, phenothiazine 5-oxide, and phenothiazine 5,5-dioxide units as a central core with the typical butterfly structure (see also Figure S17). In the case of **NPI-PTZ1** and **NPI-PTZ4**, the HOMOs are localized over the phenothiazine strong donor unit while LUMOs are mainly concentrated on the naphthalimide acceptor. In the case of the oxygen-functionalized systems, due to the presence of phenothiazine 5-oxide and phenothiazine 5,5-dioxide weaker donor units, the HOMOs are spread over the whole molecule while the LUMOs are mainly concentrated on the 1,8-naphthalimide unit, acting as a powerful acceptor.

The theoretical HOMO–LUMO band gaps for **NPI-PTZ1**, **NPI-PTZ4**, **NPI-PTZ2, NPI-PTZ3**, and **NPI-PTZ5** are 2.75, 2.77, 3.10, 3.18, and 2.97 eV, respectively (Figure S18). The oxygen-free phenothiazine derivatives thus show a decreased band gap relative to the oxygen-functionalized ones. In order to investigate the spectroscopic properties, TD-DFT calculations were carried out in dichloromethane, and the results are shown in Table S2. The electronic absorption spectra calculated by TD-DFT are in reasonable agreement with the experimental spectra.

### Solvent Effect and Hyperpolarizability

**Figure 3:**
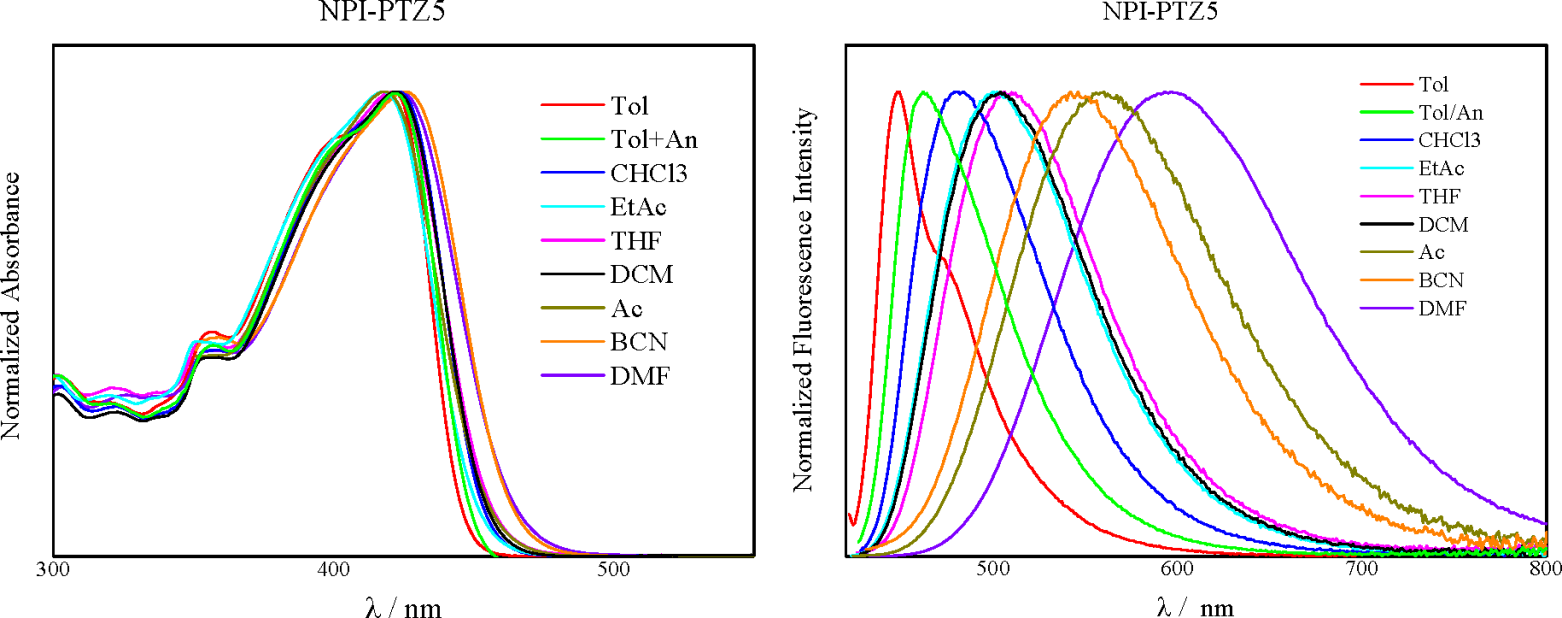
Solvent effect on the absorption (left) and emission (right) spectra of **NPI-PTZ5**.

The effect of the solvent on the absorption and emission spectra was investigated for **NPI-PTZ1–5**. One representative example is shown in Figure 3 (**NPI-PTZ5**), while all the other data are reported in detail in Figures S20–S22. The solvent effect is negligible on the absorption spectra, while being very significant on the emission spectra. For all the samples, a large red shift of the emission maximum was observed upon increasing the solvent polarity. In the case of the oxygen-free molecules, the emission spectra always appear bell-like shaped (Figures S20 and S22) and are red-shifted beyond 850 nm in the most polar media. For the oxygen-functionalized compounds, the solvent plays the role to tune the spectral shape: the fluorescence spectrum is structured in the low polar solvents but becomes broad and bell-like shaped in the more polar media (Figure 3).

The Stokes shift (Δυ) values were plotted as a function of the solvent properties (*f*(ε, n^2^)) according to the McRae equation (see Figure S23 and Table S3). From the slope of the linear fits performed on these trends, the difference between the excited- and ground-state dipole moment (Δμ) was obtained (see Table S4). The Δμ values are significant in agreement with the positive fluorosolvatochromism. In particular, Δμ is generally higher for the oxygen-free compared to the oxygen-functionalized systems (29.4, 26.9, and 26.1 D for **NPI-PTZ1**, **NPI-PTZ2**, and **NPI-PTZ3**, respectively), in line with the phenothiazine being a stronger electron donor relative to phenothiazine-5-oxide and phenothiazine-5,5-dioxide. An estimate of the frequency-dependent (β_CT_) and frequency-independent (β_0_) hyperpolarizability was obtained through the Oudar equation. For the dipolar molecules, the obtained β_0_ is roughly the same, around 60 × 10^–30^ esu^–1^ cm^5^. Enhanced hyperpolarizabilities are found for the quadrupolar molecules, particularly for the oxygen-functionalized chromophore (β_0_ = 146 × 10^–30^ esu^–1^ cm^5^ for **NPI-PTZ5** and 71.2 × 10^–30^ esu^–1^ cm^5^ for **NPI-PTZ4**). Thus, the effect of the oxygen functionalization of the phenothiazine is positive not only on the linear but also on the nonlinear optical properties of these molecules.

The effect of the solvent on the fluorescence quantum yield was also investigated (Table 2). A completely different behavior has been observed for the oxygen-free relative to the oxygen-functionalized molecules. In the case of **NPI-PTZ1** and **NPI-PTZ4**, the fluorescence quantum yield drastically decreases upon increasing the solvent polarity:^44,45^ it is reduced by 2 orders of magnitude on going from Tol to DMF. In contrast, the fluorescence efficiency is significant in all the investigated solvents for the oxygen-functionalized molecules. For **NPI-PTZ1**, an apparent viscosity effect is revealed: ϕ_F_ is 1 order of magnitude higher in the viscous BCN solvent relative to other solvents of similar polarity. This suggests that structural rearrangements may occur during excited-state deactivation.

**Table 2:** Fluorescence Quantum Yields of the Investigated Compounds in Solvents of Different Polarity and Viscosity

solvent	*f*(ε, *n*^2^)	η (cPs)	ϕ_F_ NPI-PTZ1	ϕ_F_ NPI-PTZ2	ϕ_F_ NPI-PTZ3	ϕ_F_ NPI-PTZ4	ϕ_F_ NPI-PTZ5
Tol	0.0242	0.59	0.51	0.87	0.70	0.66	0.75
Tol/An 50:50	0.143	1.16	0.20	1.27	1.16	0.47	0.73
CHCl_3_	0.293	0.58	0.017	0.92	0.90	0.12	0.76
EtAc	0.400	0.46	0.0075	0.90	0.93	0.099	0.73
THF	0.441	0.55	0.0079	0.87	0.89	0.067	0.89
DCM	0.474	0.45	0.0030	0.99	0.92	0.049	0.85
BCN	0.586	1.24	0.029	1.06	1.14	0.048	0.73
Ac	0.651	0.32	0.0015	0.50	0.90	0.047	0.68
DMF	0.664	0.92	0.0013	0.15	0.71	0.0084	0.44

### Ultrafast Spectroscopic Investigation of the Intramolecular Charge Transfer

**Figure 4:**
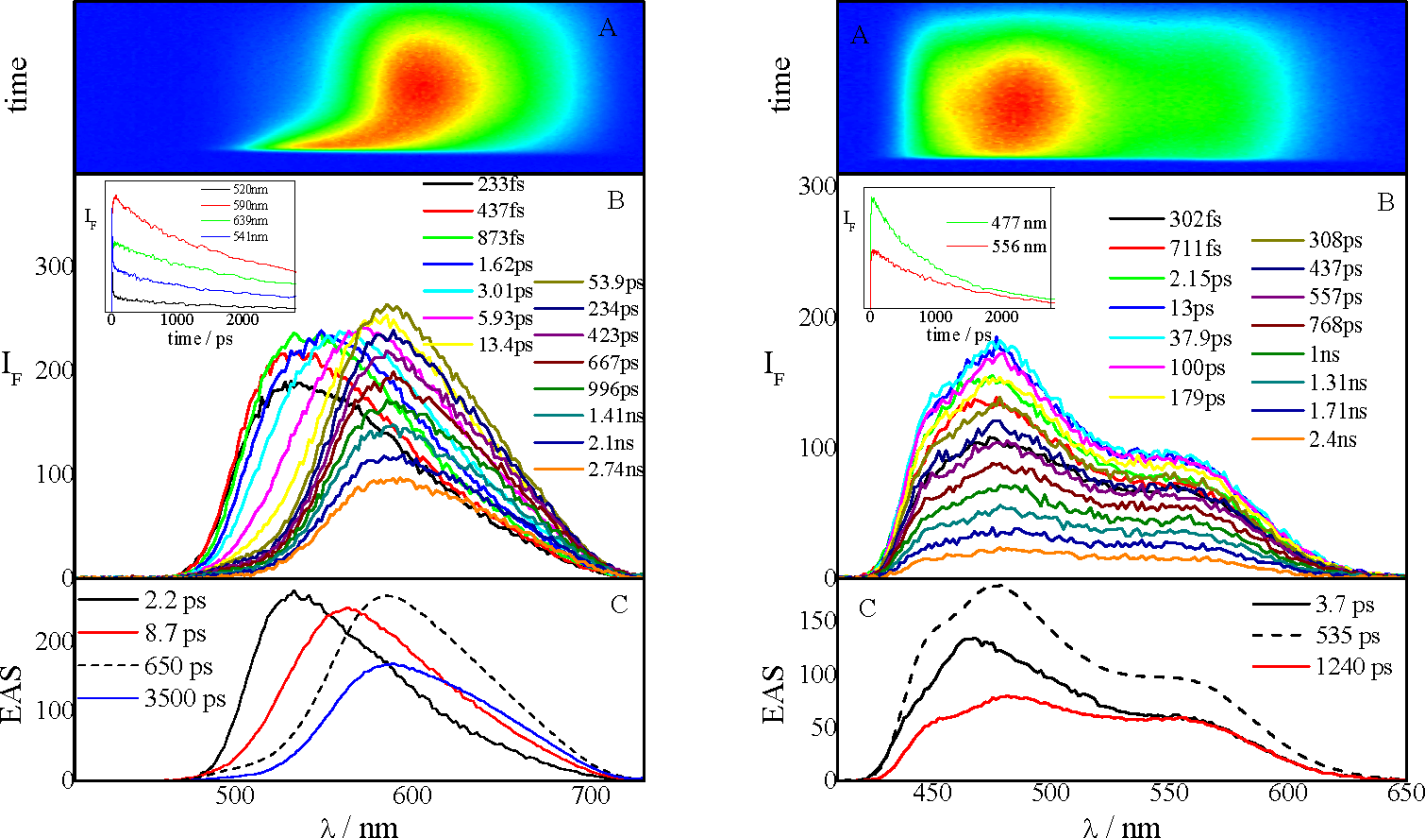
Fluorescence up-conversion spectroscopy of **NPI-PTZ4** (left) and **NPI-PTZ5** (right) in Tol.

The singlet excited-state dynamics of the NPI-PTZ molecules was investigated via femtosecond resolved spectroscopies, such as fluorescence up conversion and transient absorption. Figure 4 shows the results of the broadband fluorescence up-conversion measurements carried out in a nonpolar solvent (Tol) for the oxygen-free **NPI-PTZ4** and the oxygen-functionalized **NPI-PTZ5**, as representative examples. The exhibited behavior is very different in the two cases. For **NPI-PTZ4**, a significant red shift of the time-resolved emission spectra was observed. The emission spectrum is slightly structured right after light absorption and becomes bell-like shaped at longer delays. In the case of **NPI-PTZ5** in Tol, a structured spectrum was recorded at all delays after excitation, and its maximum does not significantly shift in time. These findings indicate that in the case of **NPI-PTZ4**, a population dynamics between two distinct excited states (the locally excited state, S_1_(LE), and an intramolecular charge-transfer state, S_1_(ICT)) may be already operative in a nonpolar solvent. On the other hand, for **NPI-PTZ5**, only the S_1_(LE) state is involved in the excited-state deactivation in Tol. Details about the results of the global analysis are given in panel C of Figure 4 and in Table S5. The transient assignments are confirmed by the transient absorption measurements carried out for the same molecules in Tol (Figure S24 and Table 3). Whereas for **NPI-PTZ4** an evolution in time of the transient absorption spectra is observed, the spectra obtained for **NPI-PTZ5** do not change their shape but just show a decay with time. In the case of **NPI-PTZ4**, the spectral shape recorded right after excitation evolves to give a transient spectrum characterized by two ESA bands at ca. 540 and 750 nm. This spectral shape is similar to that reported in the literature for the phenothiazine radical cation absorption.^46−48^ These results confirm that the phenothiazine is a stronger electron donor unit relative to phenothiazine 5-oxide and phenothiazine 5,5-dioxide, so that an ICT is observed even in a nonpolar medium for the PTZ derivatives. The transient absorption measurements have also revealed the population of a long-lived transient species (Inf) associated with the lowest excited triplet state (T_1_) (Table 3). Analogous results have been obtained for the oxygen-free and oxygen-functionalized dipolar molecules in Tol, and the results are collected in Tables 3 and S5.

**Table 3:**
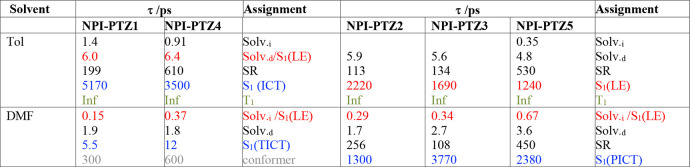
Results of Global Analysis of the Femtosecond Transient Absorption Data for the Investigated Compounds in Tol and DMF^a^

a Solv._i_ and Solv._d_, inertial and diffusive solvation, respectively; SR, structural relaxation.

**Figure 5:**
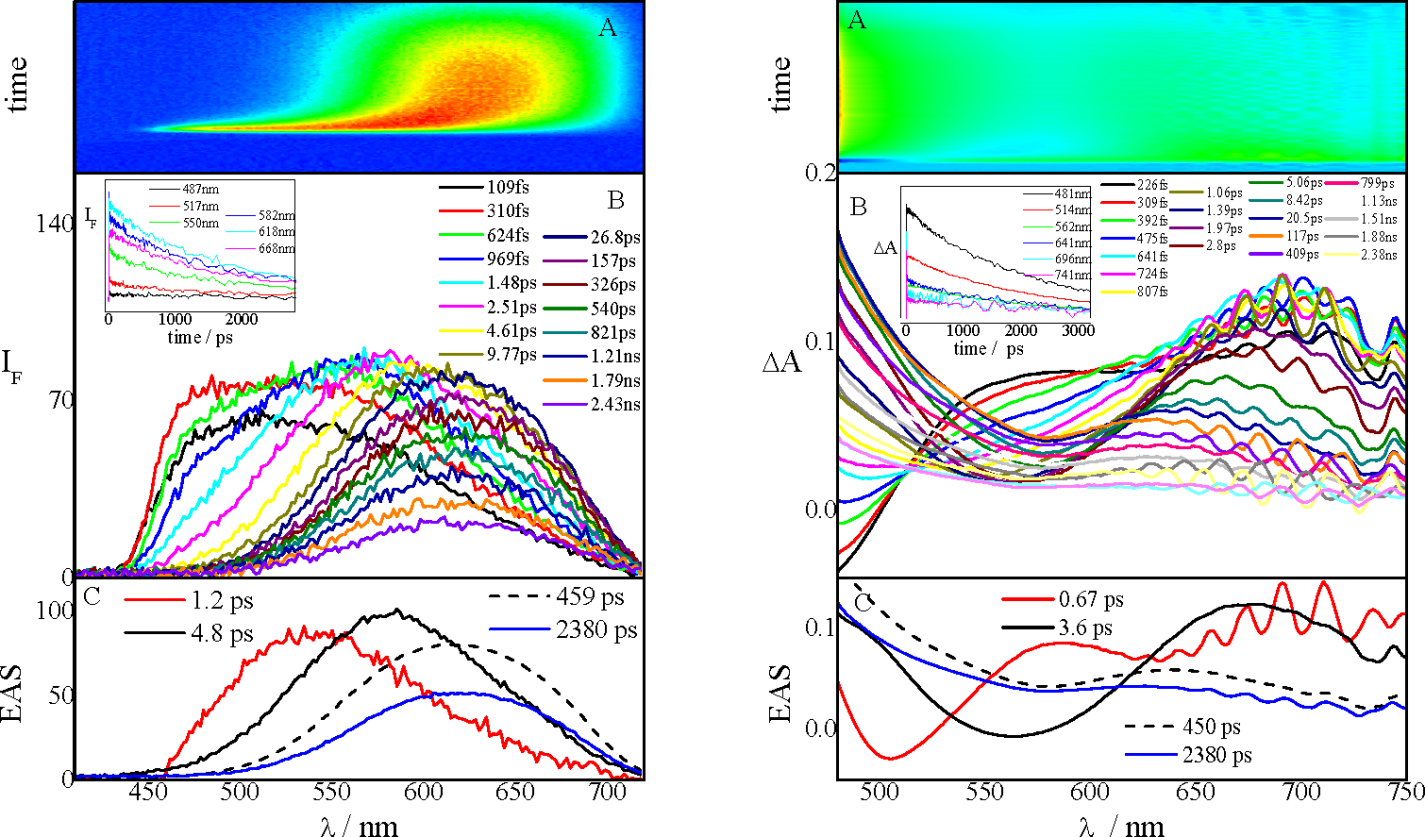
Femtosecond fluorescence up-conversion (left) and transient absorption (right) spectroscopy of **NPI-PTZ5** in DMF.

**Chart 2:**
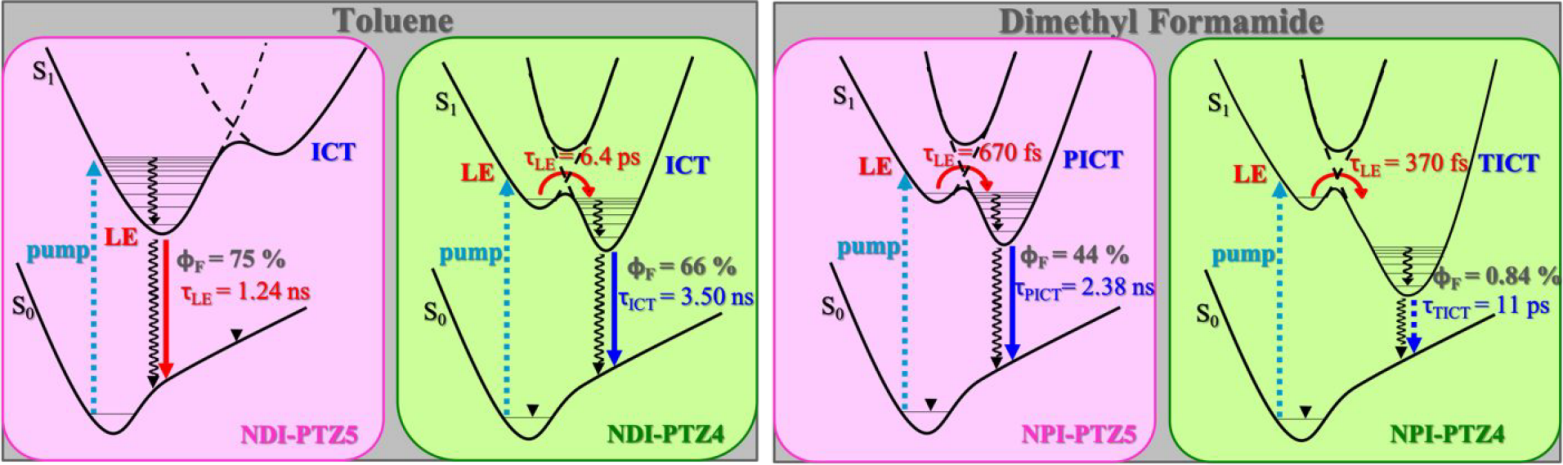
Sketch of the Excited-State Dynamics of Oxygen-Functionalized (**NPI-PTZ5**) and Oxygen-Free (**NPI-PTZ4**) Molecules in Nonpolar and Polar Solvents

In all cases, an evolution in time of both the transient emission and absorption spectra is found in a more polar solvent. This is clearly shown in Figure 5 for the case of **NPI-PTZ5** in DMF, as a representative example (see also Figure S25). The S_1_(LE) to S_1_(ICT) population dynamics takes place within the inertial solvation in a polar solvent (Table 3). For the case of **NPI-PTZ4** and **NPI-PTZ5**, the ultrafast measurements were carried out in several solvents of different polarity (Table S6), and the ICT rate solvent dependence was analyzed in the context of the Marcus theory (see Table S7 and Figure S26).^49^ A detailed analysis of the results in Table 3 allows discussion of another important difference in the behavior of the oxygen-free versus the oxygen-functionalized compounds. The S_1_(ICT) lifetime is long in Tol (a few nanoseconds) and becomes extremely short in DMF for the oxygen-free derivatives (5.5 and 11 ps for **NPI-PTZ1** and **NPI-PTZ4**, respectively). On the other hand, the lifetime revealed for the S_1_(ICT) state of the oxygen-functionalized molecules remains long in DMF (1.3–3.8 ns). This difference suggests a twisted intramolecular charge-transfer nature (TICT) of the relaxed excited state for the oxygen-free molecules, which are relatively more flexible. Differently, the S_1_(ICT) state produced for the oxygen-functionalized molecules in polar solvents should show a planar structure (PICT) in each of the butterfly branches, as suggested by the long lifetimes and significant fluorescence quantum yields. Therefore, the oxygen functionalization changes the nature of the populated ICT state (TICT for oxygen-free and PICT for oxygen-functionalized compounds) with important consequences for the emission features (see Chart 2).

**Figure 6:**
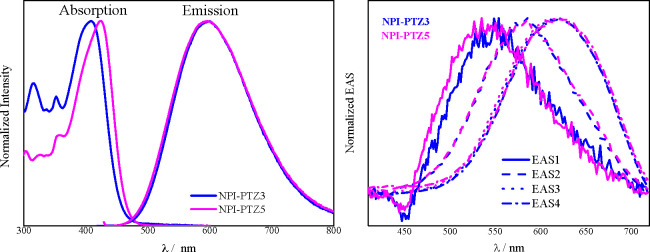
Comparison between the steady-state absorption and emission spectra (left) and between the EAS obtained by global analysis of the fluorescence up-conversion data (right) of **NPI-PTZ3** and **NPI-PTZ5** in DMF.

It is also interesting to compare the behavior of analogous dipolar and quadrupolar systems. The absorption spectra of the quadrupolar derivatives are generally red-shifted relative to those of the dipolar analogues. The emission spectra are structured and different in Tol; however, they show a surprising coincidence in polar solvents such as DMF, as shown in Figure 6 for the case of the phenothiazine-dioxide derivatives. This result suggests the occurrence of excited-state symmetry breaking (ESSB) for the quadrupolar compound in polar solvents.^50−53^ The broadband fluorescence up-conversion measurements give a deep insight into the dynamics of this ESSB (Figures 6 and S27). The analogous spectral evolution in time observed for the phenothiazine-dioxide dipolar and quadrupolar derivatives in DMF indicates that this SB is ultrafast for **NPI-PTZ5**, within the occurrence of inertial solvation (evolution associated spectra, EAS1 in Figure 6).

### Triplet Properties

**Figure 7:**
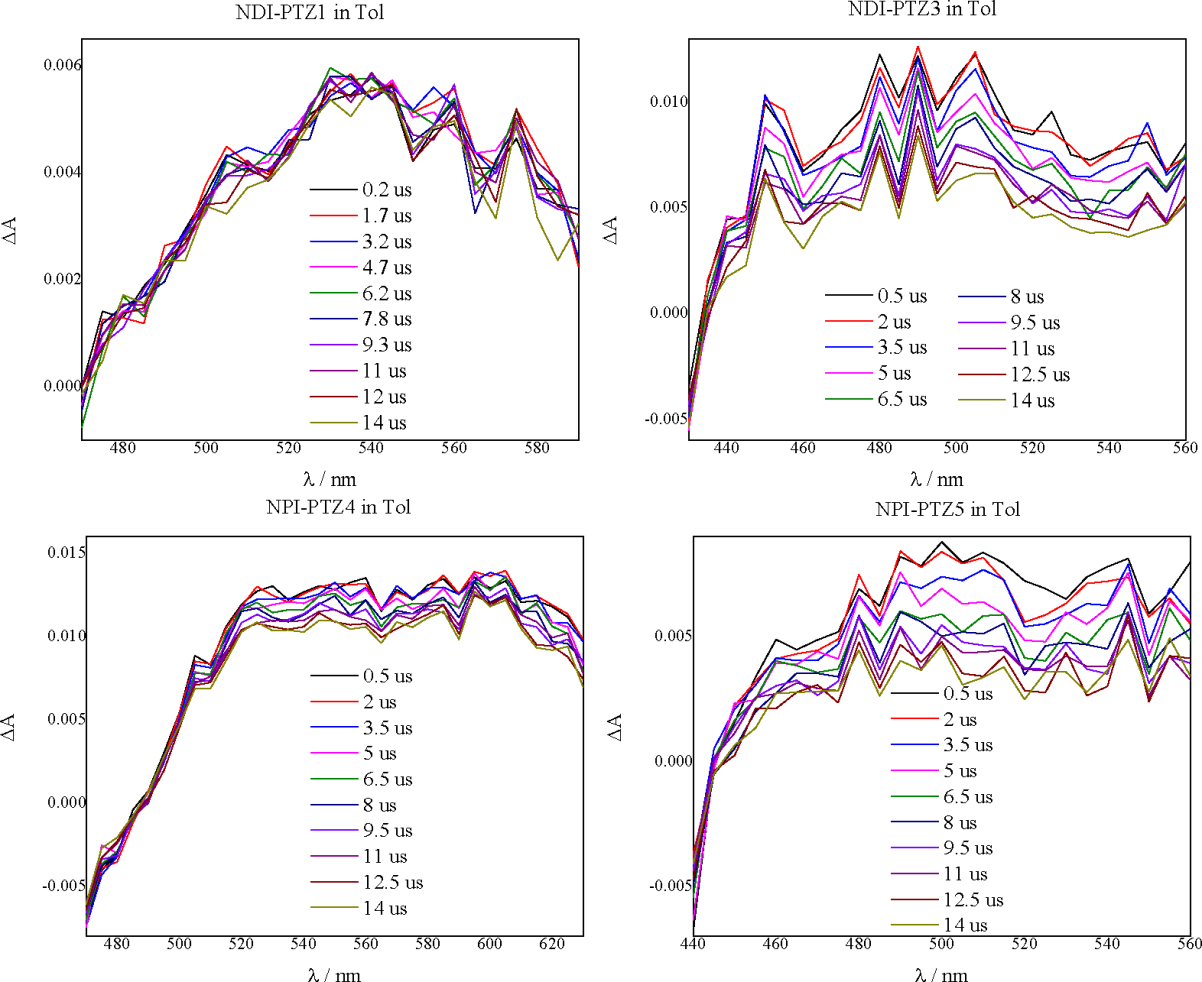
Nanosecond transient absorption spectra recorded for the investigated compounds in nitrogen purged toluene.

The triplet excited-state dynamics was investigated via nanosecond transient absorption. The obtained transient spectra in Tol are shown in Figure 7 (**NPI-PTZ2** is not shown for the lower signal/noise ratio). The transient absorption maximum of the broad positive band detected is at ca. 535 nm for **NPI-PTZ1**, and it is blue-shifted for the phenothiazine-oxide and dioxide monobranched derivatives (490 nm). The absorption band is slighthly red-shifted when passing to the quadrupolar analogues (600 nm for **NPI-PTZ4** and 500 nm for **NPI-PTZ5**). The lifetime of this transient species is hundreds of nanoseconds in air-equilibrated solution and tens of microseconds in nitrogen-purged solutions (Table 4). The same transient absorption signals could be produced by energy transfer in sensitization experiments where 2,2′-dithienyl ketone (DTK)^54−56^ was employed as the high-energy triplet donor and **NPI-PTZ1**/**NPI-PTZ3** were employed as the triplet energy acceptors (see Figures S28–S30). The oxygen effect on the lifetime and the sensitization experiments demonstrate that the revealed transient absorption is relative to the T_1_ state. The triplet lifetime (τ_T,N2_ in Table 4) decreases with the oxygen functionalization (65 μs for **NPI-PTZ1**, 34 μs for **NPI-PTZ2**, and 25 μs for **NPI-PTZ3**) and in the quadrupolar structures (42 μs for **NPI-PTZ4** and 33 μs for **NPI-PTZ5**).

**Table 4:** Triplet Properties Obtained by Nanosecond Transient Absorption^a^

compound	solvent	λ_T_ (nm)	τ_T,air_ (ns)	τ_T,N_2__ (μs)	ϕ_T_	ϕ_Δ_
**NPI**(56)	Tol	410	370	9.3	0.95	1.12
**NPI-PTZ1**	Tol	535	266	65		0.31
**NPI-PTZ2**	Tol	490		34		0.15
**NPI-PTZ3**	Tol	490	372	25		0.31
**NPI-PTZ4**	Tol	600	230	42		0.19
**NPI-PTZ5**	Tol	500	168	33		0.22

a Singlet oxygen quantum yields (ϕ_Δ_) obtained by means of phosphorescence measurements employing phenalenone in Tol (ϕ_Δ_ = 0.99) as a reference compound.

The involvement of the triplet excited state in the deactivation has also been investigated by means of singlet oxygen phosphorescence measurements (Figure S31). These experiments allowed quantitative determination of the singlet oxygen quantum yields (ϕ_Δ_), which may be considered estimates of the triplet yields (ϕ_T_). The ϕ_Δ_ values measured in Tol are reported in Table 4, together with the yield measured for the parent NPI compound, whose ϕ_T_ was previously obtained through triplet sensitization.^57^ The good agreement between the NPI triplet (ϕ_T_ = 0.95) and singlet oxygen (ϕ_Δ_ = 1.12) yields, within the experimental error, indicates the reliability of our method. The ϕ_Δ_ obtained for the NPI-PTZ compounds in Tol are between 19 and 31%. These values show a trend consistent with the ϕ_F_ in Tol (Table 1). For the oxygen-functionalized compounds, we find that ϕ_F_ + ϕ_Δ_ ≈ 1; this suggests that the excited-state deactivation in a nonpolar solvent is justified considering just the fluorescence and intersystem crossing. For the oxygen-free compounds, ϕ_F_ + ϕ_Δ_ < 1; this points to a role played by internal conversion to the ground state from the ICT state even in a nonpolar medium.

## Conclusions

We have designed and synthesized 1,8-naphthalimide-based phenothiazine derivatives, both with dipolar and quadrupolar structures, in which we have increased the oxidation state of the sulfur atom on the phenothiazine unit by one or two oxygen functionalizations. Our results show that the oxygen substitution as well as the quadrupolar structural motif have a positive impact on the optical (absorption extinction coefficient and fluorescence efficiency) and nonlinear optical (hyperpolarizability) properties of these new organic materials. The nanosecond time-resolved spectroscopic experiments revealed a certain involvement of the lowest triplet excited state in the deactivation (intersystem crossing) of these compounds. The electrochemical study demonstrates that the phenothiazine 5-oxide and phenothiazine 5,5-dioxide show reduced electron-donating ability relative to the phenothiazine unit. The quantum chemical simulations predicted HOMOs localized on the phenothiazine strong donor unit for the oxygen-free derivatives and delocalized over the whole molecular structure for the oxygen-functionalized derivatives, with the LUMOs being localized on the naphthalimide strong acceptor in all cases. The ultrafast spectroscopic measurements, transient absorption, and fluorescence up conversion uncovered the detailed intramolecular charge-transfer mechanism, subtly tuned by the molecular structure of these push–pull chromophores. In particular, a planar intramolecular charge transfer (PICT) and thus highly fluorescent state was populated upon photoexcitation of the sulfoxide and sulfone-based compounds in polar solvents. Differently, the relaxed singlet state exhibits a twisted intramolecular charge transfer (TICT) nature in the case of the oxygen-free phenothiazines, leading to significant fluorescence quenching. The advanced broadband fluorescence up-conversion spectroscopy data suggest that the photoinduced ICT occurs by breaking the excited-state symmetry in the quadrupolar chromophores. Our results show that synthetically tuning the sulfur oxidation state in these molecules leads to either highly efficient intramolecular charge transfer (phenothiazine derivatives) or highly efficient emission (phenothiazine-oxide and dioxide derivatives). These findings establish oxygen functionalization as a new effective synthetic strategy to tailor the photophysics of phenothiazine-based organic materials for different optoelectronic applications. The new materials here thoroughly investigated for their optical and photophysical properties are thus promising for either photon-to-current conversion applications in organic photovoltaics (oxygen-free compounds) or current-to-photons applications in organic light-emitting diodes (oxygen-functionalized compounds).

## Supporting Information Available

Additional data about the synthesized sample characterization; theoretical calculations; cyclic voltammetry results; spectral, photophysical, and ultrafast spectroscopic results (PDF)
