# Supplementary material for: Tuning the Fluorescence and the Intramolecular Charge Transfer of Phenothiazine Dipolar and Quadrupolar Derivatives by Oxygen Functionalization
Source: J Am Chem Soc. 2021 Jun 23;143(26):9933–43. doi: 10.1021/jacs.1c04173 (PMC8297855; doi:10.1021/jacs.1c04173)
Supplement: Supplementary file 1 — ja1c04173_si_001.pdf [file ja1c04173_si_001.pdf]

Supplementary Information

Tuning the Fluorescence and the Intramolecular Charge Transfer of Phenothiazine Dipolar and Quadrupolar Derivatives by Oxygen Functionalization

Yogajivan Rout,[1] Chiara Montanari,[2] Erika Pasciucco,[2] Rajneesh Misra,[*,1] Benedetta Carlotti[*,2]

[1]Department of Chemistry, Indian Institute of Technology, Indore 453552, India. E-mail: rajneeshmisra@iiti.ac.in

[2]Department of Chemistry, Biology and Biotechnology, University of Perugia, via elce di sotto 8, 06123 Perugia, Italy. E-mail: benedetta.carlotti@unipg.it

Table of Contents

Experimental Section. ··················· **S3**

Figures S1–S15. ^{1}H NMR, ^{13}C NMR spectra and HRMS for **NPI-PTZ1– NPI-PTZ15**··················· **S9**

Figure S16. Cyclic voltammograms of **NPI-PTZ1–5**··················· **S24**

Table S1. Electrochemical data ··················· **S25**

Theoretical calculations for **NPI-PTZ1– NPI-PTZ5** ··················· **S25**

Figure S17. Lateral view of the ground state optimized geometries and dihedral angles for the investigated compounds. ··················· **S32**

Table S2. Electronic absorption transitions for the all molecules in dichloromethane··········· **S33**

Figure S18. Energy levels diagram of the frontier orbitals estimated by DFT calculations······ **S33**

Figure S19. Molar extinction coefficients of the investigated compounds in Tol·············· **S34**

Figure S20. Solvent effect on the absorption (left) and emission (right) spectra of **NPI-PTZ4** ··················· **S34**

Figure S21 and S22 Solvent effect on the absorption and emission spectra of the dipolar compounds. ··················· **S35**

Table S3. Spectral properties of the investigated compounds. ··················· **S36**

Figure S23. Plot of the Stokes shift as a function of the solvent properties for the investigated compounds.··················· **S38**

Table S4. Hyperpolarizability coefficients derived by the fluorosolvatochromism of the investigated compounds ··················· **S38**

Figure S24. Femtosecond transient absorption spectroscopy of **NPI-PTZ4** (left) and **NPI-PTZ5** (right) in Tol. ··················· **S39**

Figure S25. Femtosecond fluorescence up conversion (left) and transient absorption (right) spectroscopy of **NPI-PTZ4** in DMF. ··················· **S39**

Table S5. Results of the global fitting of the femtosecond transient absorption (TA) and fluorescence up conversion (FUC) data. ··················· **S40**

Table S6. Results of the global fitting of the femtosecond transient absorption data of **NPI-PTZ4** and **NPI-PTZ5** in several solvents of increasing polarity. ··················· **S41**

Table S7. Charge transfer rate constant (k_{et}) and charge transfer free energy variation (ΔG_0), evaluated for **NPI-PTZ4** and **NPI-PTZ5** from the experimental data. ··························**S41**

Figure S26. Dependence of the charge transfer rate constant (k_{et}) upon the charge transfer free energy variation (ΔG_0) in the context of the Marcus theory······································**S42**

Figure S27. Femtosecond fluorescence up conversion spectroscopy of **NPI-PTZ3** in DMF. ···**S42**

Figure S28. Triplet absorption spectra and decay kinetics obtained for DTK in MeCN by nanosecond transient absorption. ···**S43**

Figure S29. Triplet absorption spectra and decay kinetics obtained during the sensitization experiment carried out by employing DTK as triplet energy donor and **NPI-PTZ1** as triplet energy acceptor in MeCN through nanosecond transient absorption. ·····································**S44**

Figure S30. Triplet absorption spectra and decay kinetics obtained during the sensitization experiment carried out by employing DTK as triplet energy donor and **NPI-PTZ3** as triplet energy acceptor in MeCN through nanosecond transient absorption. ·····································**S44**

Figure S31. Phosphorescence spectra of the singlet oxygen produced by photoexcitation of the investigated compounds in Tol. ··**S45**

Experimental Section

Synthesis and Characterization: Chemicals were used as received unless otherwise indicated. All the oxygen or moisture sensitive reactions were carried out under argon atmosphere. ^{1}H NMR spectra were recorded using a 400 MHz spectrometer. Chemical shifts are reported in delta (δ) units, expressed in parts per million (ppm) downfield from tetramethylsilane (TMS) using residual protonated solvent as an internal standard {$CDCl_3$, 7.26 ppm}. ^{13}C NMR spectra were recorded using a 100 MHz spectrometer. Chemical shifts are reported in delta (δ) units, expressed in parts per million (ppm) downfield from tetramethylsilane (TMS) using the solvent as internal standard {$CDCl_3$, 77.16 ppm}. The ^{1}H NMR splitting patterns have been described as "s, singlet; d, doublet; t, triplet and m, multiplet". UV-visible absorption spectra of all compounds were recorded in dichloromethane solution. Cyclic voltammograms and differential pulse voltammograms were recorded on potentiostat using glassy carbon as working electrode, Pt wire as the counter electrode, and saturated calomel electrode (SCE) as the reference electrode. The scan rate was 100 mVs^{-1} for cyclic voltammetry. A solution of tetrabutylammonium hexafluorophosphate ($TBAPF_6$) in DCM (0.1M) was used as supporting electrolyte.

Synthesis of NPI-PTZ1

In a 100 mL round bottomed flask 3-ethynyl-10-propyl-10H-phenothiazine **2** (0.132 g, 0.5 mmol) and 6-bromo-2-butyl-1H benzo[de]isoquinoline-1,3(2H)-dione **1** (0.166 g, 0.5 mmol) were dissolved in 1:1 (v/v) triethylamine (TEA) (30 ml) and tetrahydrofuran (THF) (30ml) . The

reaction mixture was purged with argon, and $Pd(PPh_3)_4$ (0.041 g, 0.071 mmol), and CuI (0.007 g, 0.078 mmol) were added. The reaction mixture was reflux for 6 h. Upon the completion of the reaction, the mixture was evaporated and purified by silica gel column chromatography with hexane/CH_2Cl_2 (1:1) to get the desired compound **NPI-PTZ1** as a red colored solid. Yield 0.155 g (60%); ^{1}H NMR (400 MHz, $CDCl_3$): δ 8.71 (d, J=8 Hz, 1H), 8.65 (d, J=8 Hz, 1H), 8.55 (d, J=8 Hz, 1H), 7.91 (d, J=8 Hz, 1H), 7.83 (t, 1H), 7.45–7.41 (m, 2H), 7.19–7.13 (m, 2H), 6.95 (t, 1H), 6.87 (t, 2H), 4.19 (t, 2H), 3.85 (t, 2H), 1.90–1.85 (m, 2H), 1.75–1.73 (m, 2H), 1.49–1.43 (m, 2H), 1.05–0.96 (m, 6H); ^{13}C NMR (100 MHz, $CDCl_3$): δ 164.21, 163.94, 146.56, 144.45, 132.53, 131.68, 130.57, 128.25, 127.99, 127.67, 127.46, 125.27, 124.09, 123.17, 121.92, 115.85, 115.30, 99.18, 86.61, 49.54, 40.46, 30.36, 20.54, 20.24, 14.00, 11.42; MALDI-TOF-MS: m/z: $[M]^+$ calcd. for $C_{33}H_{28}N_2O_2S$: 516.1871; found 516.2803.

Synthesis of NPI-PTZ4

In a 100 mL round bottomed flask 3,7-diethynyl-10-octyl-10H-phenothiazine derivative **3** (0.179 g, 0.5 mmol) and 6-bromo-2-butyl-1H-benzo[de]isoquinoline-1,3(2H)-dione **1** (0.331 g, 1 mmol) were dissolved in 1:1 (v/v) triethylamine (TEA) (30 ml) and tetrahydrofuran (THF) (30ml) . The reaction mixture was purged with argon, and $Pd(PPh_3)_4$ (0.041 g, 0.071 mmol), and CuI (0.007 g, 0.078 mmol) were added. The reaction mixture was reflux for 6 h. Upon the completion of the reaction, the mixture was evaporated and purified by silica gel column chromatography with hexane/CH_2Cl_2 (1:3) to get the desired compound **NPI-PTZ4** as a red colored solid. Yield 0.273 g (63%); ^{1}H NMR (400 MHz, $CDCl_3$): δ 8.72 (d, J=8 Hz, 2H), 8.66 (d, J=8 Hz, 2H), 8.56 (d, J=8 Hz, 2H), 7.93 (d, J=8 Hz, 2H), 7.84 (t, 2H), 7.48 (d, J=8 Hz, 2H), 7.42 (s, 2H), 6.91 (d, J=8 Hz, 2H), 4.19 (t, 4H), 3.92 (t, 2H), 1.87–1.84 (m, 2H), 1.77–1.69 (m, 4H), 1.49–1.28 (m, 13H), 0.99 (t, 6H), 0.89–0.83 (m, 4H); ^{13}C NMR (100 MHz, $CDCl_3$): δ 164.16, 163.90, 145.44, 132.43, 131.71, 130.62, 130.52, 128.24, 127.75, 127.51, 124.36, 123.18, 122.07, 116.70, 115.59, 98.68, 86.96, 40.47, 30.36, 29.84, 29.34, 29.31, 26.95, 26.85, 20.54, 13.99; MALDI-TOF-MS: m/z: $[M]^+$ calcd. for $C_{56}H_{51}N_3O_4S$: 861.360; found 861.778.

Synthesis of NPI-PTZ2

In a 100 mL round bottomed flask m-Chloroperbenzoic acid (0.241 g, 1.4 mmol) was added to **NPI-PTZ** (0.516 g, 1 mmol) in chloroform (30 ml) at room temperature for 1h. Upon the completion of the reaction, the mixture was purified by silica gel column chromatography with CH_2Cl_2 to get the desired compound **NPI-PTZ2** as an orange colored solid. Yield 0.452 g (85%); ^{1}H NMR (400 MHz, $CDCl_3$): δ 8.76 (d, J=8 Hz, 1H), 8.67 (d, J=8 Hz, 1H), 8.58 (d, J=8 Hz, 1H), 8.27 (s, 1H), 7.98 (t, 2H), 7.89–7.85 (m, 2H), 7.68 (t, 1H), 7.47–7.43 (m, 2H), 7.32 (t, 1H), 4.26–7.18 (m, 4H), 2.07–2.01 (m, 2H), 1.77–1.70 (m, 2H), 1.51–1.42 (m, 2H), 1.17 (t, 3H) 0.99 (t, 3H); ^{13}C NMR (100 MHz, $CDCl_3$): δ 164.06, 163.79,138.63, 137.94, 135.83, 135.69, 133.32, 132.35, 131.95, 131.70, 131.60, 130.73, 130.42, 128.14, 127.60, 127.40, 124.55, 124.40, 123.12, 122.69, 122.23, 116.18, 116.15, 115.50, 97.79, 87.25, 50.04, 40.44, 30.33, 20.51, 19.88, 13.97, 11.17; HRMS (ESI-TOF) m/z $[M +H]^+$ calcd for $C_{33}H_{28}N_2O_3S$ 533.1893; measured 533.1905.

Synthesis of NPI-PTZ3

In a 100 mL round bottomed flask m-Chloroperbenzoic acid (0.517 g, 3 mmol) was added to **NPI-PTZ** (0.516 g, 1 mmol) in chloroform (30 ml) at room temperature for 1h. Upon the completion of the reaction, the mixture was purified by silica gel column chromatography with CH_2Cl_2 to get the desired compound **NPI-PTZ3** as an orange colored solid. Yield 0.427 g (78%); ^{1}H NMR (400 MHz, $CDCl_3$): δ 8.75 (d, *J*=8 Hz, 1H), 8.67 (d, *J*= 8 Hz, 1H), 8.58 (d, *J*= 8 Hz, 1H), 8.44 (s, 1H), 8.18 (d, *J*= 8 Hz, 1H), 7.98 (d, *J*=8 Hz, 1H), 7.90–7.85 (m, 2H), 7.67 (t, 1H), 7.40–7.33 (m, 3H), 4.22–7.16 (m, 4H), 2.05–1.95 (m, 2H), 1.77–1.70 (m, 2H), 1.51–1.42 (m, 2H), 1.11 (t, 3H) 0.99 (t, 3H); ^{13}C NMR (100 MHz, $CDCl_3$): δ 164.0, 163.8, 141.0, 140.5, 136.0, 133.6, 132.3, 131., 131.6, 130.9, 130.4, 128.1,127.7, 127.7, 127.1, 124.6, 123.9, 123.1, 122.7, 122.4, 116.5, 116.4, 115.8, 97.2, 87.6, 50.3, 40.5, 30.3, 20.5, 20.3, 14.0, 11.1; HRMS (ESI-TOF) m/z [M +H]$^+$calcd for $C_{33}H_{28}N_2O_4S$ 549.1848; measured 549.1847.

Synthesis of NPI-PTZ5

In a 100 mL round bottomed flask 3,7-diethynyl-10-octyl-10H-phenothiazine 5,5-dioxide derivative **5** (0.195 g, 0.5 mmol) and 6-bromo-2-(2-ethylhexyl)-1H-benzo[de]isoquinoline-1,3(2H)-dione **4** (0.388 g, 1 mmol) were dissolved in 1:1 (v/v) triethylamine (TEA) (30 ml) and tetrahydrofuran (THF) (30ml) . The reaction mixture was purged with argon, and $Pd(PPh_3)_4$ (0.041 g, 0.071 mmol), and CuI (0.008 g, 0.078 mmol) were added. The reaction mixture was reflux for 6 h. Upon the completion of the reaction, the mixture was evaporated and purified by silica gel column chromatography with hexane/CH_2Cl_2 (1:1) to get the desired compound **NPI-PTZ5** as a yellow colored solid. Yield 0.297 g (59%); ^{1}H NMR (400 MHz, $CDCl_3$): δ 8.76 (d, *J*=8 Hz, 2H), 8.69 (d, *J*=8 Hz, 2H), 8.59 (d, *J*=8 Hz, 2H), 8.47 (d, *J*=8 Hz, 2H), 8.00 (d, *J*=8 Hz, 2H), 7.92–7.88 (m, 4H), 7.45 (d, *J*=8 Hz, 2H), 4.25 (t, 2H), 4.19–4,09 (m, 4H), 1.99–1.96 (m, 4H), 1.45–1.32 (m, 26H), 0.96–0.87(m, 15H); ^{13}C NMR (100 MHz, $CDCl_3$): δ 164.48, 164.22, 140.44, 136.39, 132.29,131.94, 131.68, 131.08, 130.52, 128.26, 127.86, 127.81, 126.93, 124.81, 123.24, 122.64, 116.80, 116.77, 96.74, 88.13, 44.42, 38.09, 31.86, 30.90, 29.34, 29.31, 28.86, 26.81, 24.22, 23.23, 22.75, 14.24,10.80; MALDI-TOF-MS: m/z: [M+H]$^+$ calcd. for $C_{64}H_{67}N_3O_6S$: 1005.4751; found 1005.796.

Photophysical measurements. Spectral and photophysical measurements were investigated in several solvents: toluene (Tol), anisole (An), chloroform ($CHCl_3$), ethylacetate (EtAc), tetrahydrofuran (THF), dichloromethane (DCM), benzonitrile (BCN), acetone (Ac), dimethylformamide (DMF). A Cary 4E *Varian* spectrophotometer was used for the absorption measurements. The fluorescence spectra, corrected for the instrumental response, were measured by a FluoroMax®−4P spectrofluorimeter by *HORIBA* Scientific operated by FluorEssence™. Dilute solutions (absorbance < 0.1 at the excitation wavelength, λ_{exc}) were used for fluorimetric measurements. The fluorescence quantum yield (ϕ_F, uncertainty ± 10%) was determined at λ_{exc} corresponding to the maximum of the first absorption band. Tetracene (ϕ_F = 0.17[a] in air equilibrated cyclohexane) was used as fluorimetric standard. Singlet oxygen phosphorescence was measured through an *Edinburgh Instruments* FS5 spectrofluorimeter equipped with an InGaAs detector able to detect fluorescence in the infrared, up to 1600 nm. The singlet oxygen quantum

yields were obtained through comparison with a reference compound, Phenalenone in Tol, whose ϕ_Δ is known to be 1.[b] Fluorescence lifetimes were measured using the single photon counting method through the same *Edinburgh Instruments* FS5 spectrofluorimeter, equipped with LED sources centered at 370 nm, with a 0.2 ns temporal resolution.
The spectral properties and the lifetimes of long−lived transient species were probed by transient absorption with nanosecond time resolution. A LP980 (*Edinburgh*) system, with PMT for signal detection (PMT−LP), was coupled with a Spectra Physics Indi Nd:YAG nanosecond pulsed laser at 355 nm as excitation source. For this investigation, the 355 nm excitation beam was used to pump the samples and a pulsed xenon lamp white light continuum was used to probe the absorption properties of the produced excited states. The transient spectra were obtained by monitoring the change of absorbance over the 300–700 nm range. Sensitization experiments were carried out where 2,2'-dithienyl ketone (DTK)[c,d,e] was employed as the high energy triplet donor and **NPI-PTZ1**/**NPI-PTZ3** were employed as the triplet energy acceptors. MeCN was used as the solvent for these experiments as hydrogen abstraction may be observed when DTK is used in Tol.[d]
The experimental setups for femtosecond transient absorption and fluorescence up–conversion measurements have been widely described elsewhere.[f,g] In particular, the 400 nm excitation pulses of ca. 40 fs are generated by an amplified Ti:Sapphire laser system (Spectra Physics). The transient absorption set up (Helios, *Ultrafast Systems*) is characterized by temporal resolution of ca. 150 fs and spectral resolution of 1.5 nm. Probe pulses are produced in the 450–800 nm range by passing a small portion of 800 nm light through an optical delay line (with a time window of 3200 ps) and focusing it into a 2 mm thick Sapphire window to generate a white–light continuum. In the up–conversion set–up (Halcyone, Ultrafast Systems) the 400 nm pulses excite the sample whereas the remaining fundamental laser beam plays the role of the "optical gate" after passing through a delay line. The fluorescence of the sample is collected and focused onto a BBO crystal together with the delayed fundamental laser beam. A CCD detects the up–converted fluorescence. Movement of the crystal through a rotational stage allows for broadband detection of the emission at each delay and thus acquisition of the entire time–resolved fluorescence spectra. The temporal resolution of the up–conversion equipment is about 250 fs, whereas the spectral resolution is 1 nm. Ultrafast spectroscopic data were fitted by Global and Target Analysis using Surface Explorer and Glotaran softwares.[h]

Hyperpolarizability determination. The method used to derive the hyperpolarizability coefficient has been described in detail in refs. [i,j,k]. Shortly, in the case of the dipolar molecules the experimental results on solvatochromism allowed information on the difference between the excited and ground state dipole moments ($\Delta\mu = \mu_e - \mu_g$) to be obtained by using eq. 1, as derived on the basis of McRae's theory and where the solvent function $f(\varepsilon,n^2)$ is put equal to $\left(\frac{\varepsilon-1}{\varepsilon+2} - \frac{n^2-1}{n^2+2}\right)$:

$$\bar{\nu}_{abs} - \bar{\nu}_{em} = \left(\delta_{abs} + \delta_{em}\right) + \frac{2\Delta\mu_{CT}^2}{hca^3}\left(\frac{\varepsilon-1}{\varepsilon+2} - \frac{n^2-1}{n^2+2}\right) \quad (1)$$

where $\bar{\nu}_{abs} - \bar{\nu}_{em}$ is the Stokes shift (in cm^{-1}), δ_{abs} and δ_{em} are the differences in the vibrational energy (in cm^{-1}) of the molecule in the excited and ground state for absorption and emission, respectively, *h* is the Planck's constant (in erg × s), *c* is the speed of light in vacuum (in cm s^{-1}), *a* is the cavity radius within Onsager's model (in cm), ε is the relative dielectric constant and *n* the static refractive index of the solvent. The *a* value was estimated as 60% of the maximum calculated diameter of the optimized structures computed by Density Functional Theory simulations (Gaussian09) in toluene at B3LYP/6-31G (d.p) level.
In the case of the quadrupolar molecules the following equations are used:

$$\bar{\nu}_{abs} - \bar{\nu}_{em} = (\delta_{abs} - \delta_{em}) + \frac{2(Q_e - Q_g)^2}{hca^5}\left(\frac{\varepsilon - 1}{\varepsilon + 2} - \frac{n^2 - 1}{n^2 + 2}\right) \quad (2)$$

being Q = 2μd

$$(Q_e - Q_g)^2 = (2\mu_e d - 2\mu_g d)^2 = 4d^2(\mu_e - \mu_g)^2 = 4d^2\Delta\mu^2 \quad (3)$$

to obtain the difference of the quadrupole moments ($Q_e - Q_g$) and of the dipole moment of half the molecule (Δμ) considering the quadrupole as two opposite dipoles separated by a distance d, sharing the electron poor DPP group.
The hyperpolarizability was then calculated by the Oudar equation:

$$\beta_{CT} = \beta_{zzz} = \frac{3}{2h^2c^2} \times \frac{\bar{\nu}_{eg}^2 r_{eg}^2 \Delta\mu_{CT}}{(\bar{\nu}_{eg}^2 - \bar{\nu}_L^2)(\bar{\nu}_{eg}^2 - 4\bar{\nu}_L^2)} \quad (4)$$

where r_{eg} is the transition dipole moment, $\bar{\upsilon}_{eg}$ is the transition frequency (assumed to be the maximum of the bathochromic absorption band) and $\bar{\upsilon}_L$ is the frequency of the reference incident radiation to which the β value would be referred (1064 nm of the NdYAG laser usually employed for spectroscopic experiments, see below, for comparison purposes). The r_{eg} value is related to the oscillator strength (f) by:

$$r_{eg}^2 = \frac{3e^2h}{8\pi^2mc} \times \frac{f}{\bar{\nu}_{eg}} = 2.13 \times 10^{-30} \times \frac{f}{\bar{\nu}_{eg}} \quad (5)$$

(f being obtained from the absorption integrated band as $f = 4.32 \times 10^{-9} \int \varepsilon(\bar{\upsilon})\, d\bar{\upsilon}$).
It has to be noted that despite the methods based on the solvent effect on the spectra contain several approximations, thus allowing only an approximate estimation of β, a good agreement has been reported for several compounds between the β values thus estimated and those experimentally determined by more refined techniques (the well−known Electric Field Induced Second Harmonic, EFISH, and Hyper-Rayleigh Scattering, HRS) and theoretically calculated. The solvatochromic methods offer the advantage of simplicity and easy availability on EFISH generation. Being based on the production of the charge transfer states in polar solvents, the method here used gives the β_{CT} dominant contribution (corresponding to the β_{xxx} component of the β tensor when related to the charge transfer transition).
The static hyperpolarizability, whose value is instead frequency independent, can be defined as follows:

$$\beta_0 = \frac{3}{2h^2c^2} \times \frac{r_{eg}^2 \Delta\mu}{\upsilon_{eg}^2} \tag{6}$$

a. Birks, J. B. Photophysics of Aromatic Molecules; Wiley- Interscience: London, 1970; p 123.
b. Schmidt, R.; Tanielian, C.; Dunsbach, R.; Wolff, C. Phenalenone, a Universal Reference Compound for the Determination of Quantum Yields of Singlet Oxygen Sensitization. *J. Photochem. Photobiol. A: Chem,* **1994**, 79, 11–17.
c. Ciorba, S.; Carlotti, B.; Škorić, I.; Šindler−Kulyk, M.; Spalletti, A. "Spectral properties and photobehaviour of 2,5−distyrylfuran derivatives"; *J. Photochem. Photobiol. A: Chemistry* **2011**, *219*, 1−9.
d. Ortica, F.; Romani, A.; Favaro, G. "Light-Induced Hydrogen Abstraction from Isobutanol by Thienyl Phenyl, Dithienyl, and Thienyl Pyridyl Ketones" *J. Phys. Chem. A*, **1999**, *103*, 1335−1341.
e. Becker, R.S.; Favaro, G.; Poggi, G.; Romani, A. "Photophysical Properties of Some Thienyl Ketones: An Experimental and Theoretical Study" *J. Phys. Chem.*, **1995**, *99*, 1410–1417.
f. Ricci, F.; Carlotti, B.; Keller, B.; Bonaccorso, C.; Fortuna, C. G.; Goodson, T.; Elisei, F.; Spalletti, A. Enhancement of Two-Photon Absorption Parallels Intramolecular Charge-Transfer Efficiency in Quadrupolar versus Dipolar Cationic Chromophores. *J. Phys. Chem. C*, **2017**, *121*, 3987–4001.
g. Carlotti, B.; Benassi, E.; Spalletti, A.; Fortuna, C. G.; Elisei, F.; Barone, V. Photoinduced symmetry-breaking intramolecular charge transfer in a quadrupolar pyridinium derivative. *Phys. Chem. Chem. Phys.*, **2014,** *16*, 13984–13994.
h. Snellenburg, J. J.; Laptenok, S.; Seger, R.; Mullen, K. M.; van Stokkum, I. H. M. Glotaran: A Java−Based Graphical User Interface for the R Package TIMP. *J. Stat. Soft*. **2012**, 49 (3), 1−22.
i. Carlotti, B.; Flamini, R.; Kikaš, I.; Mazzucato, U.; Spalletti, A. Intramolecular charge transfer, solvatochromism and hyperpolarizability of compounds bearing ethenylene or ethynylene bridges. *Chem. Phys.* **2012**, *407*, 9–19.
j. Carlotti, B.; Cesaretti, A.; Cacioppa, G.; Elisei, F.; Odak, I.; Škorić, I.; Spalletti, A. Fluorosolvatochromism and hyperpolarizability of one-arm and two-arms nitrocompounds bearing heterocyclic rings. *J. Photochem. Photobiol. A: Chem.*, **2019**, *368*, 190–199.
k. F. Ricci, F. Elisei, P. Foggi, A. Marrocchi, A. Spalletti and B. Carlotti, "Photobehavior and Nonlinear Optical Properties of Push−Pull, Symmetrical, and Highly Fluorescent Benzothiadiazole Derivatives", *J. Phys. Chem. C,* 2016, **120**, 23726−23739.

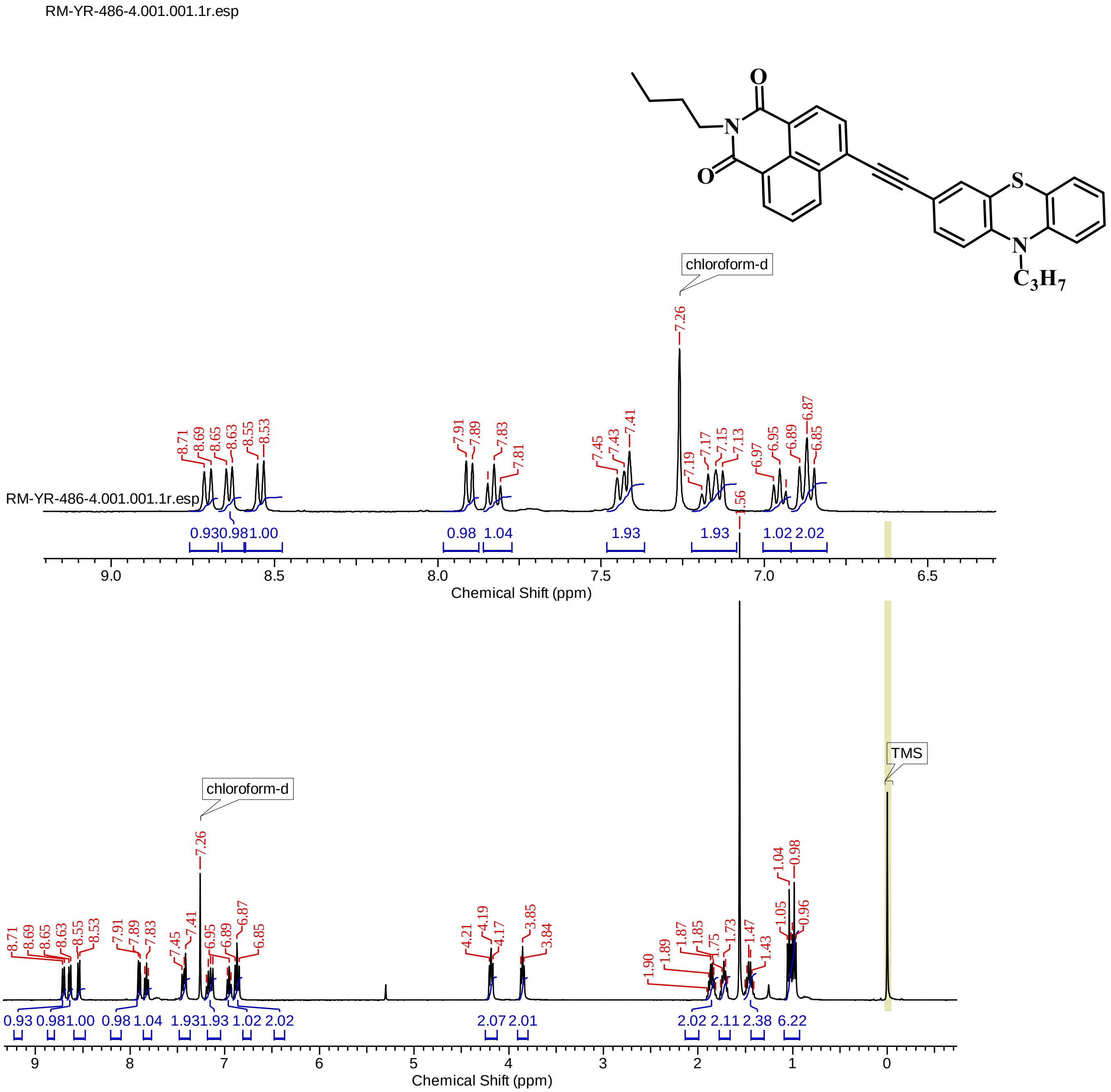

Figure S1. ^{1}H NMR spectra of **NPI-PTZ1.**

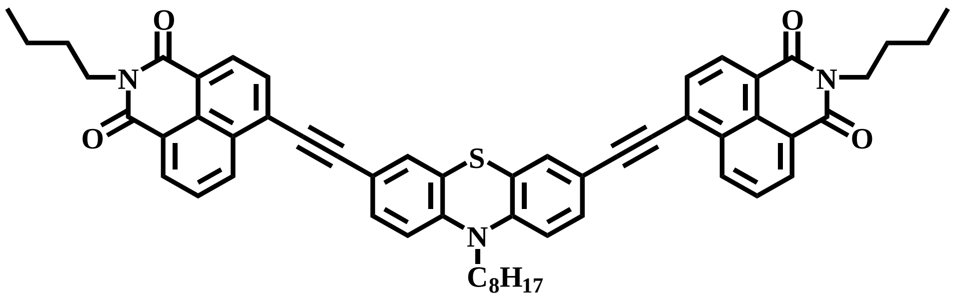

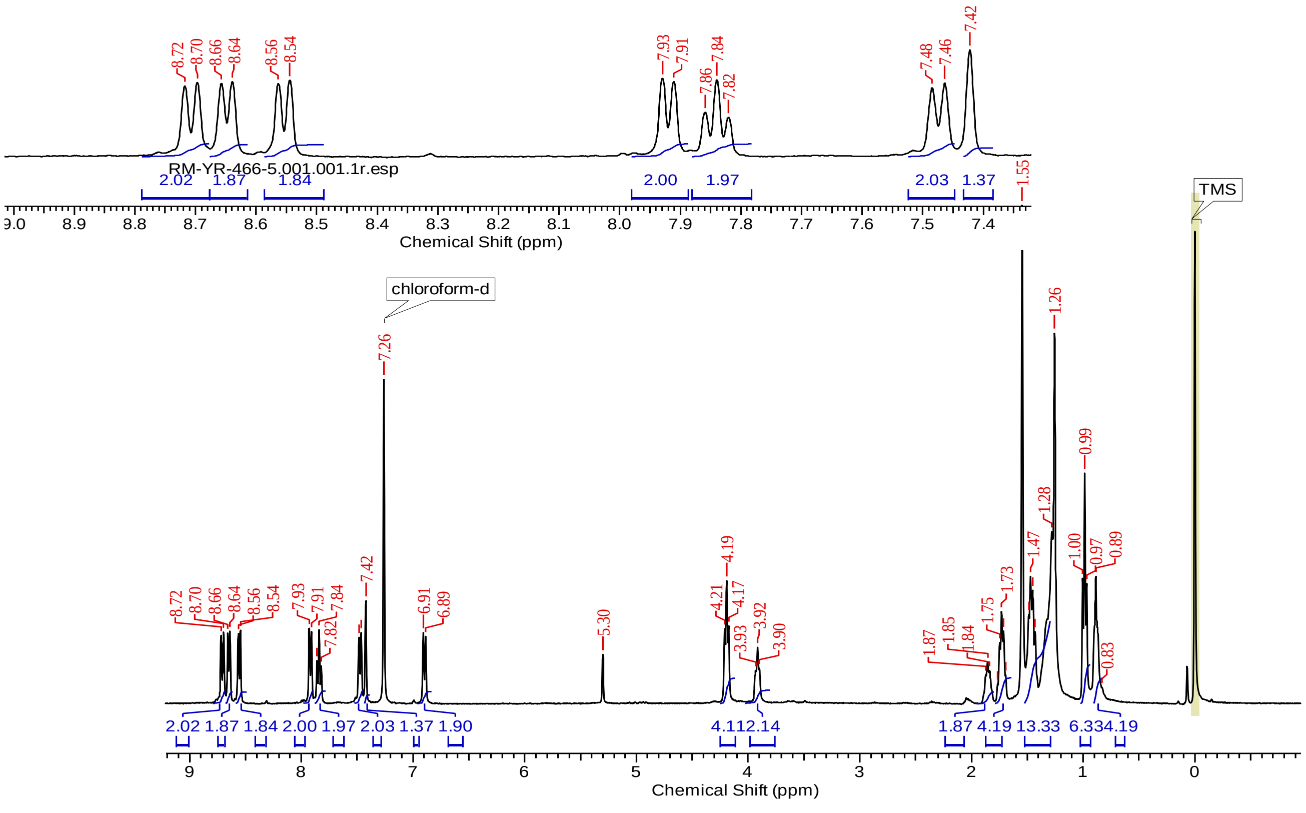

Figure S2. ^{1}H NMR spectra of **NPI-PTZ4.**

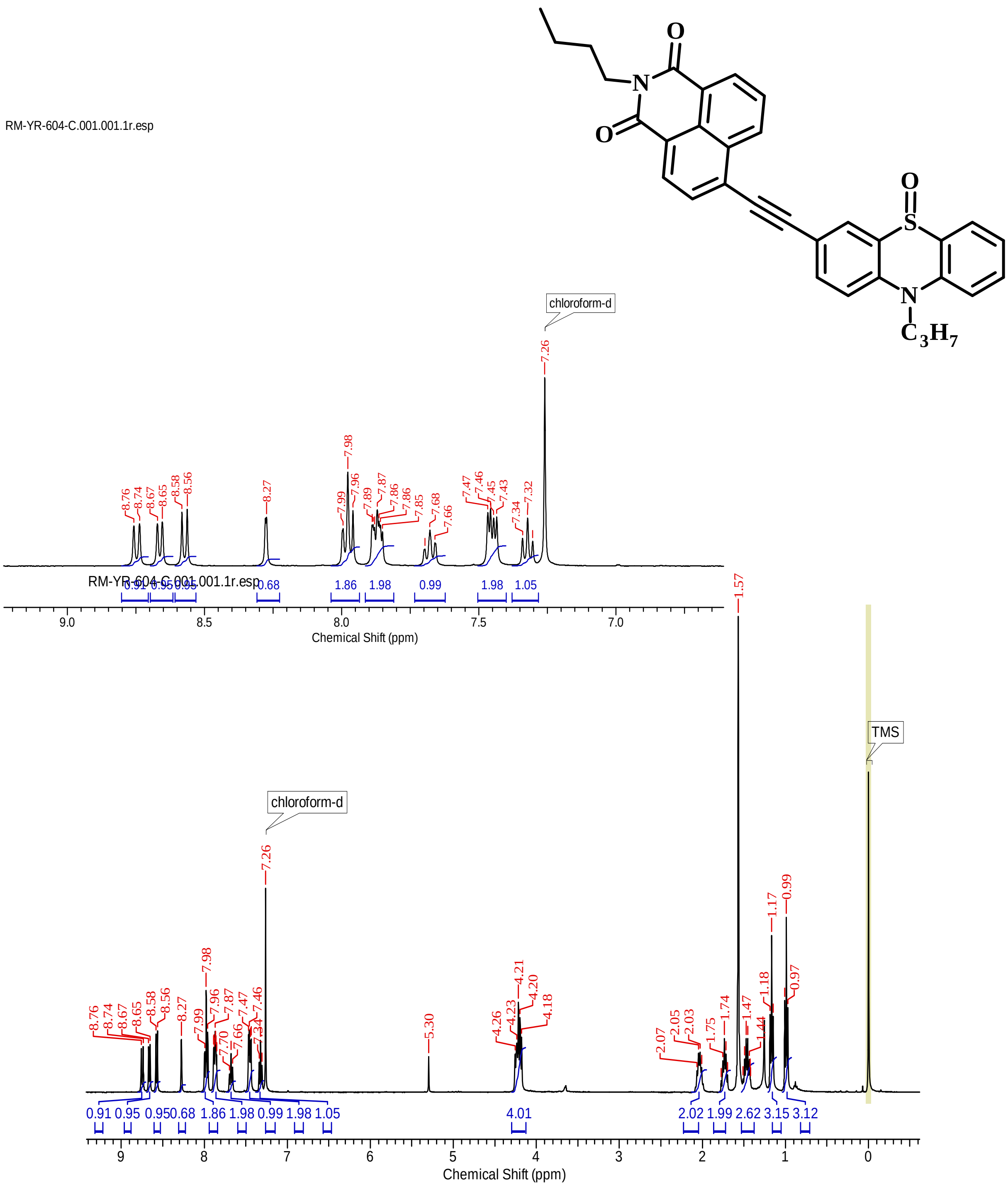

Figure S3. ^{1}H NMR spectra of **NPI-PTZ2.**

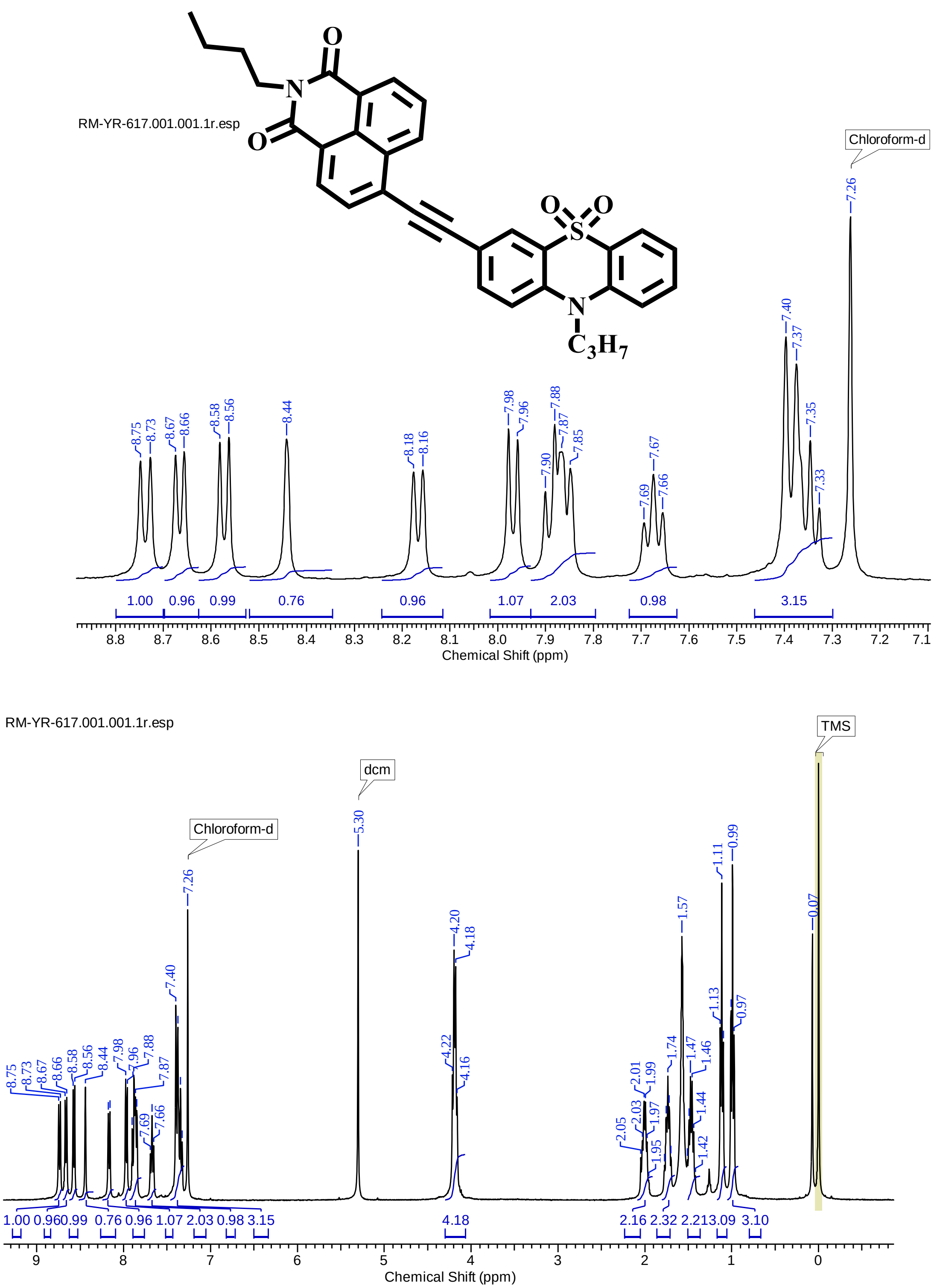

Figure S4. ^{1}H NMR spectra of **NPI-PTZ3.**

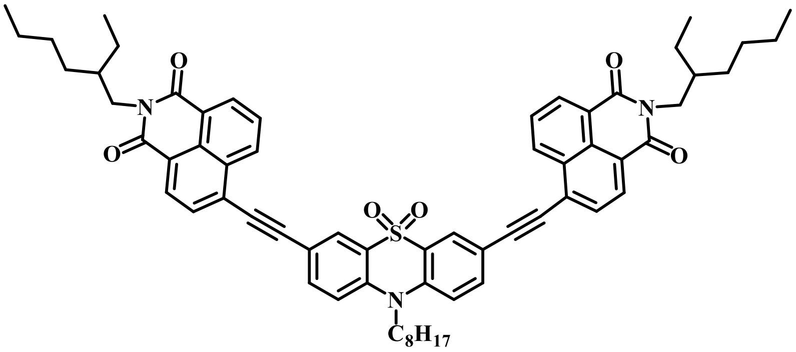

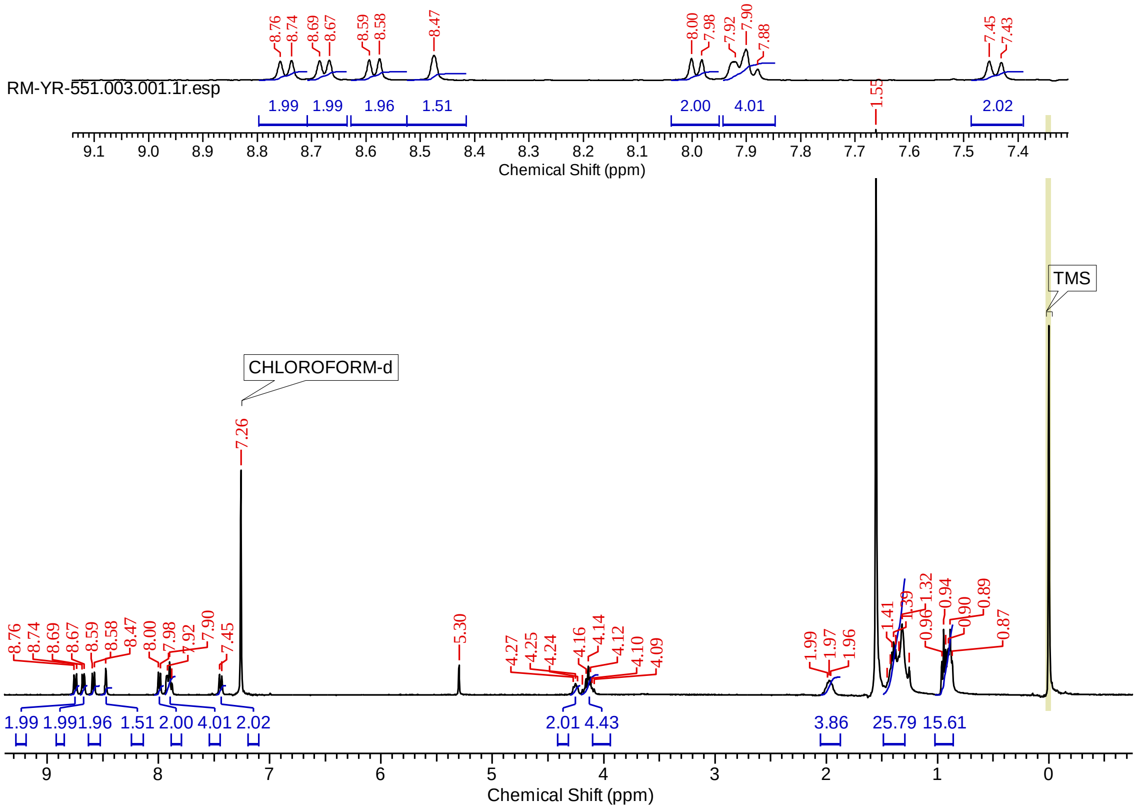

Figure S5. ^{1}H NMR spectra of **NPI-PTZ5.**

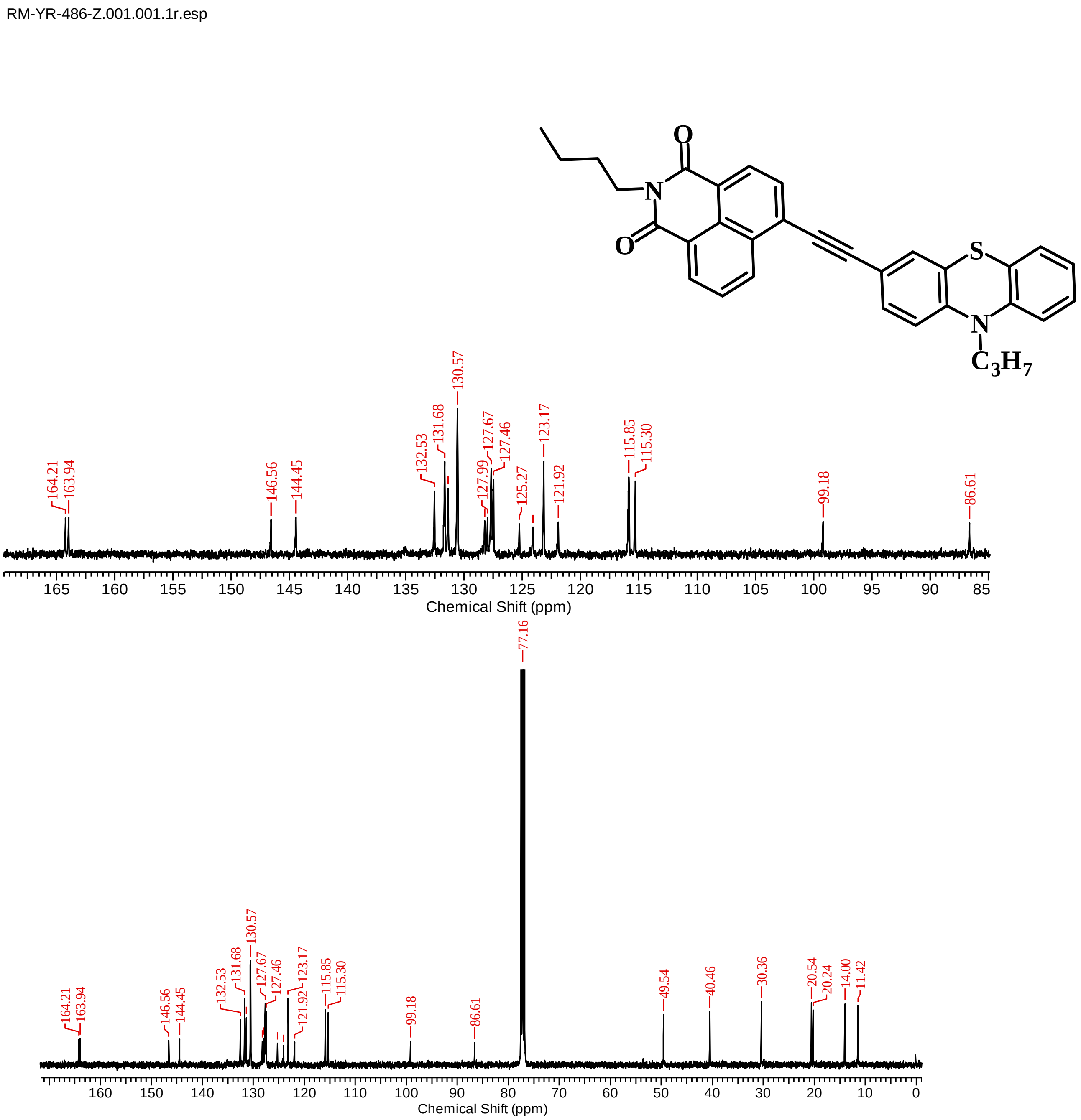

Figure S6. ^{13}C NMR spectra of **NPI-PTZ1.**

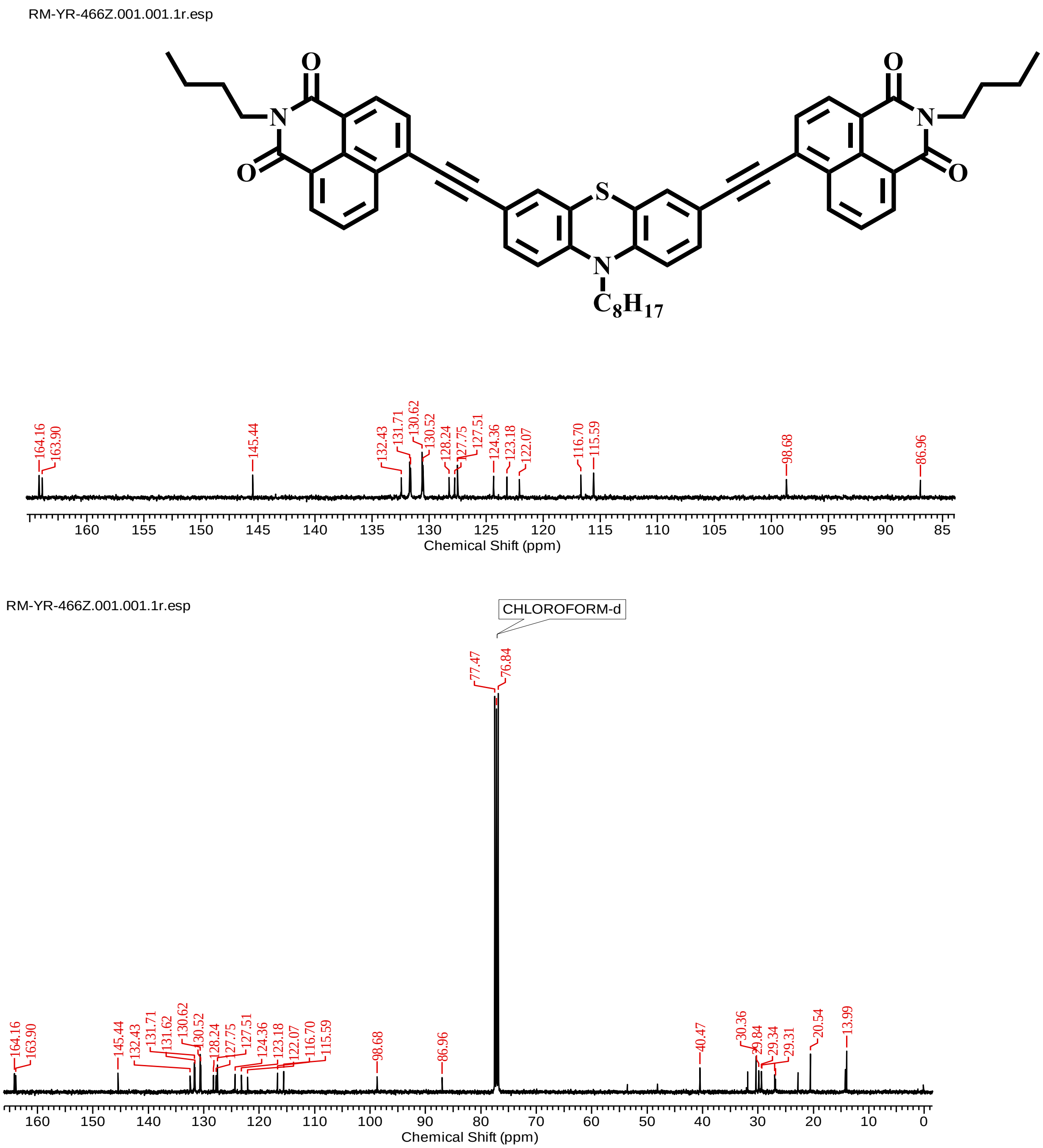

Figure S7. ^{13}C NMR spectra of **NPI-PTZ4.**

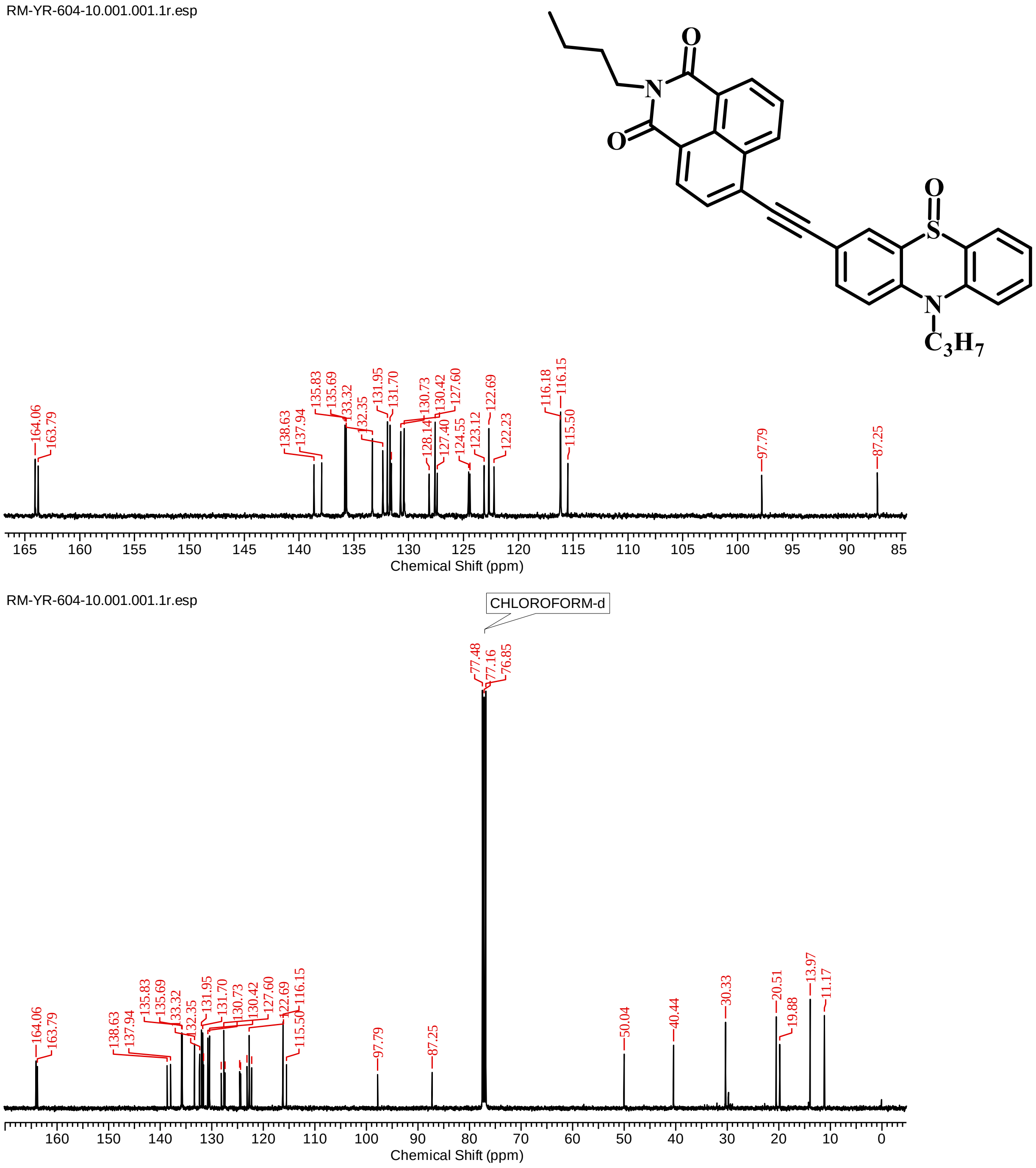

Figure S8. ^{13}C NMR spectra of **NPI-PTZ2.**

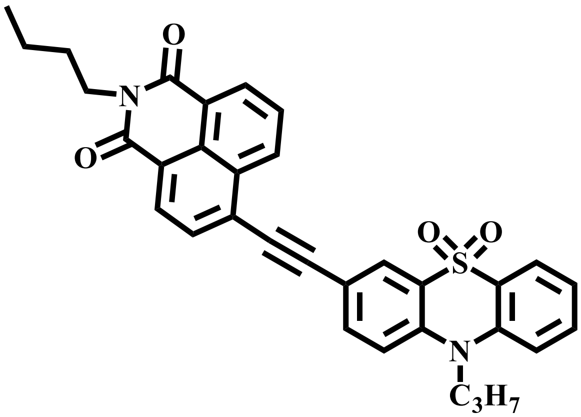

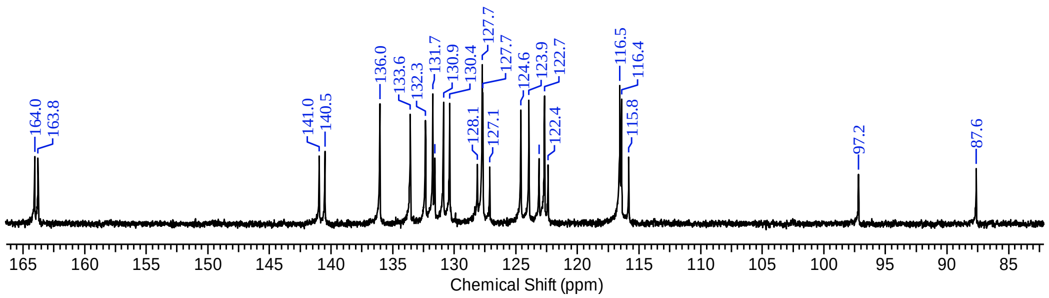

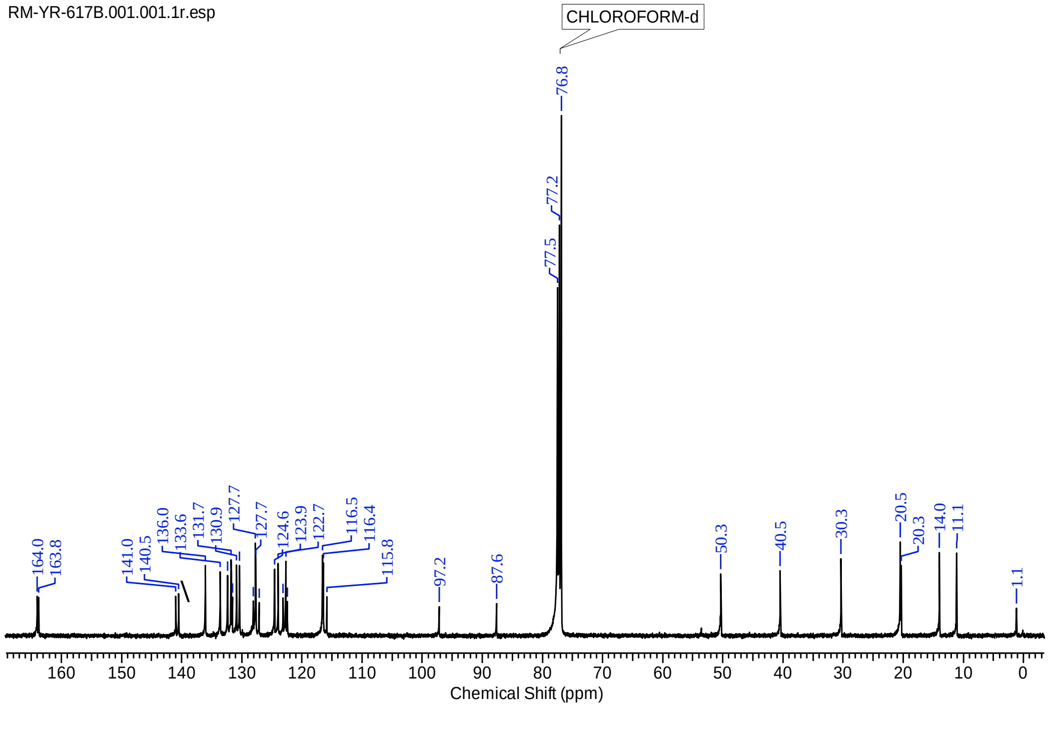

Figure S9. ^{13}C NMR spectra of **NPI-PTZ3.**

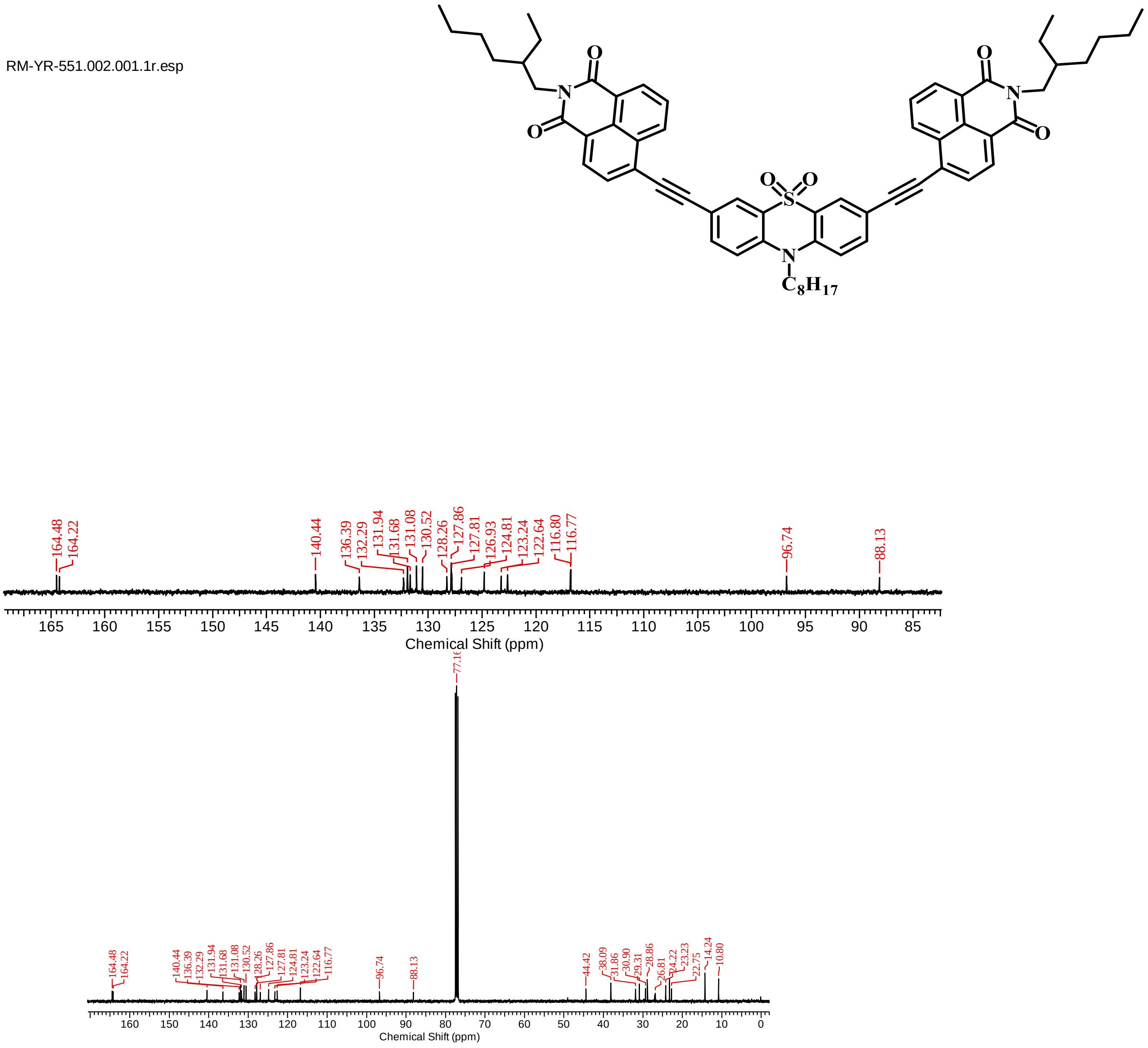

Figure S10. ^{13}C NMR spectra of **NPI-PTZ5.**

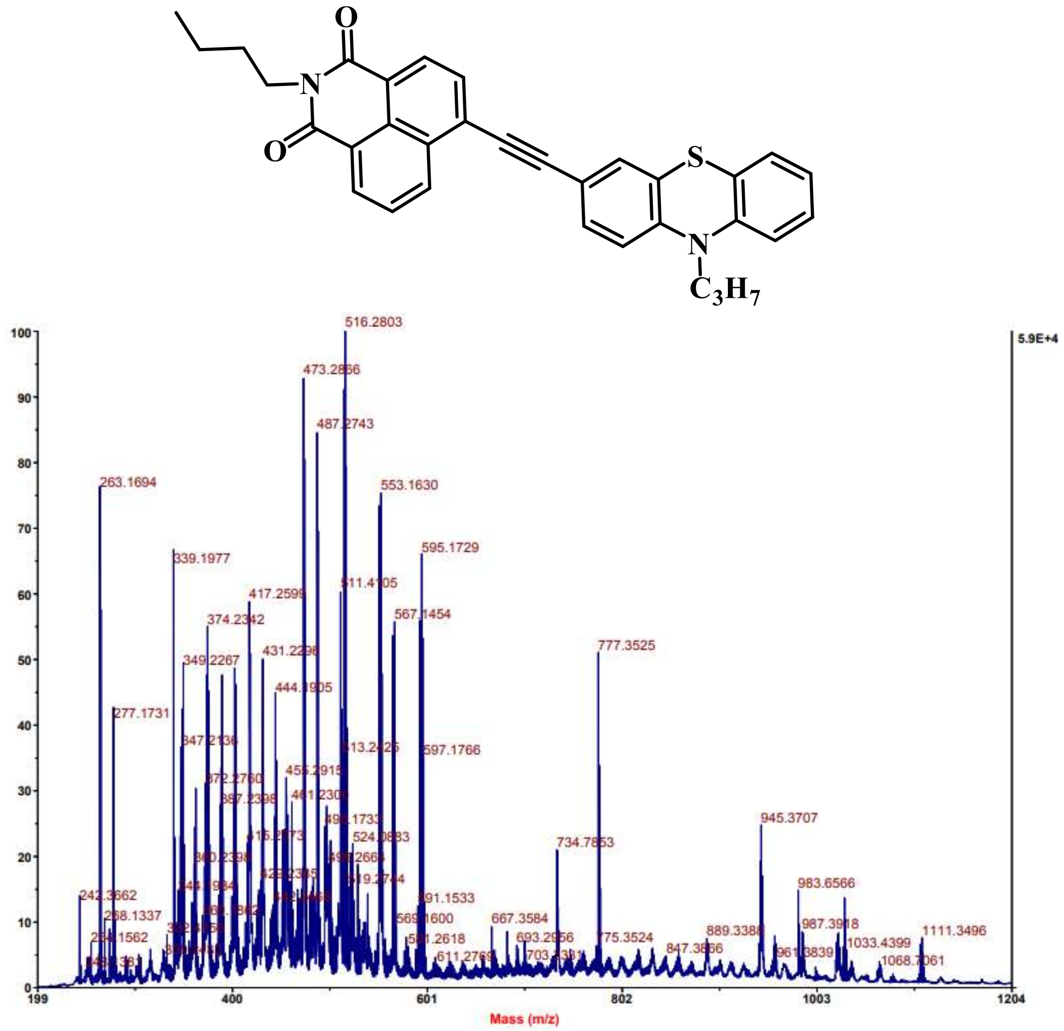

Figure S11. MALDI spectra of **NPI-PTZ1**.

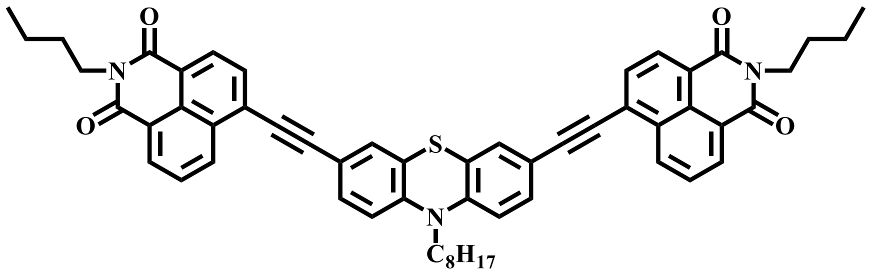

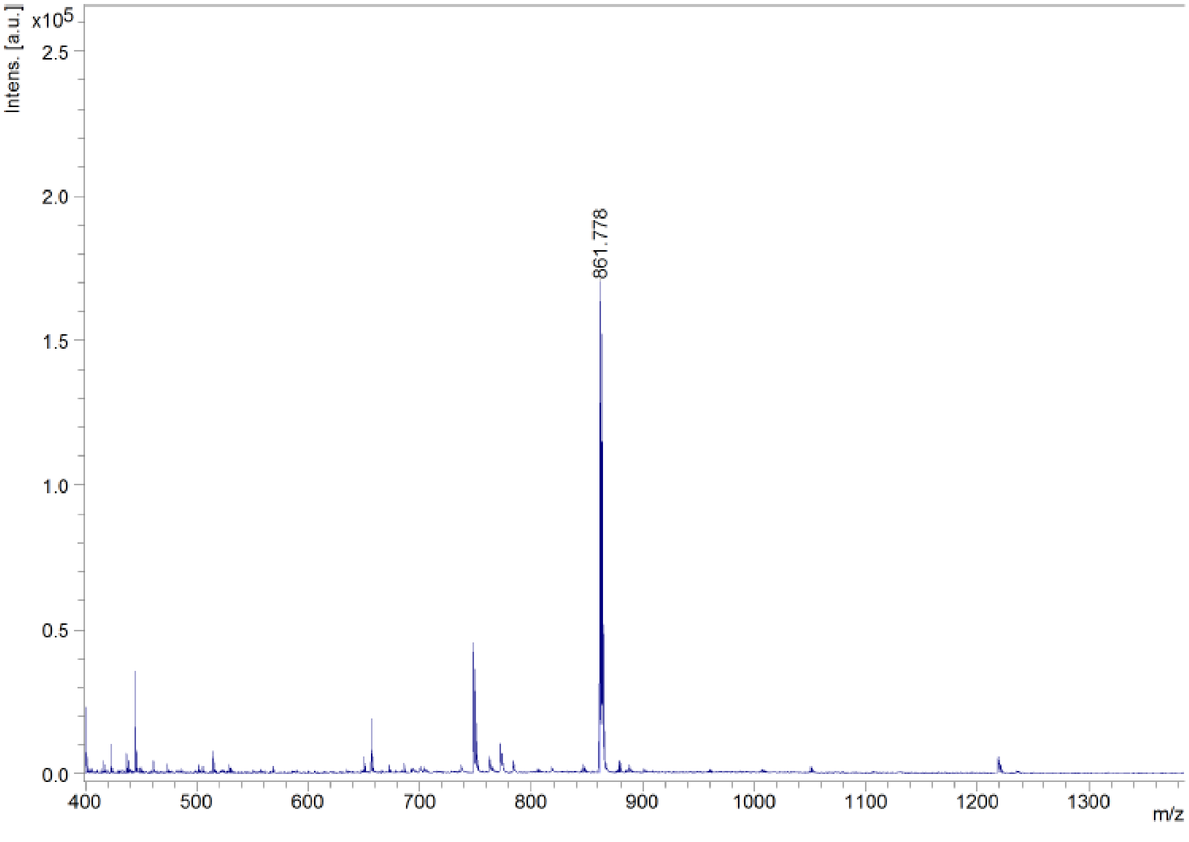

Figure S12. MALDI spectra of **NPI-PTZ4**.

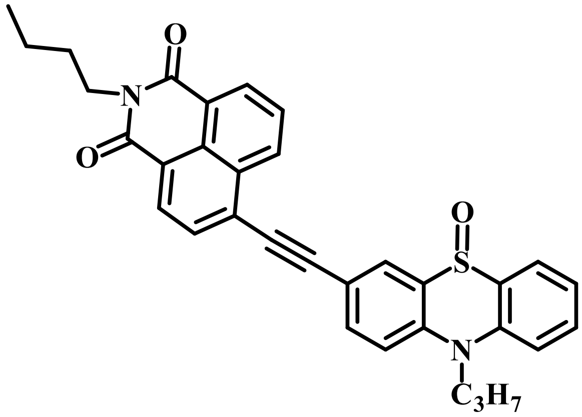

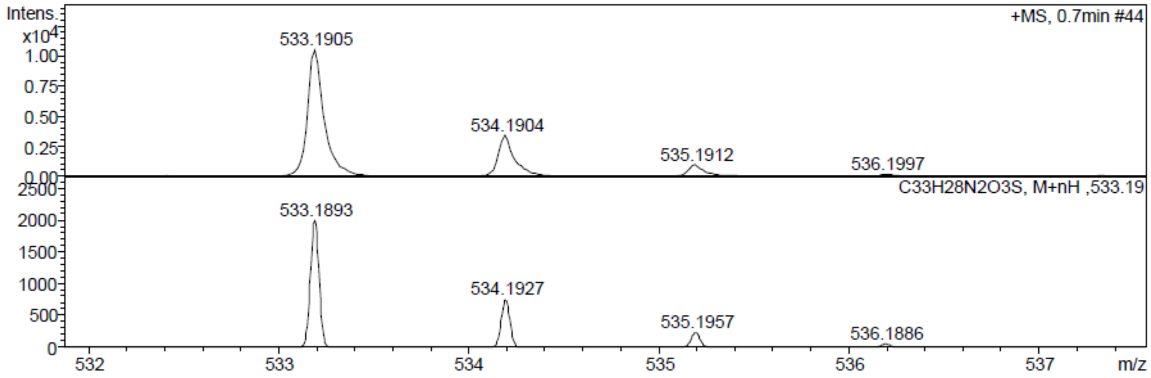

Figure S13. HRMS of **NPI-PTZ2.**

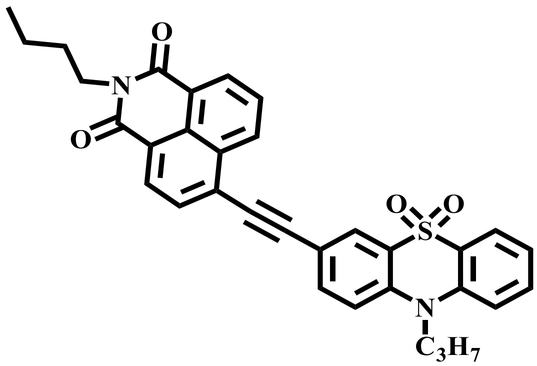

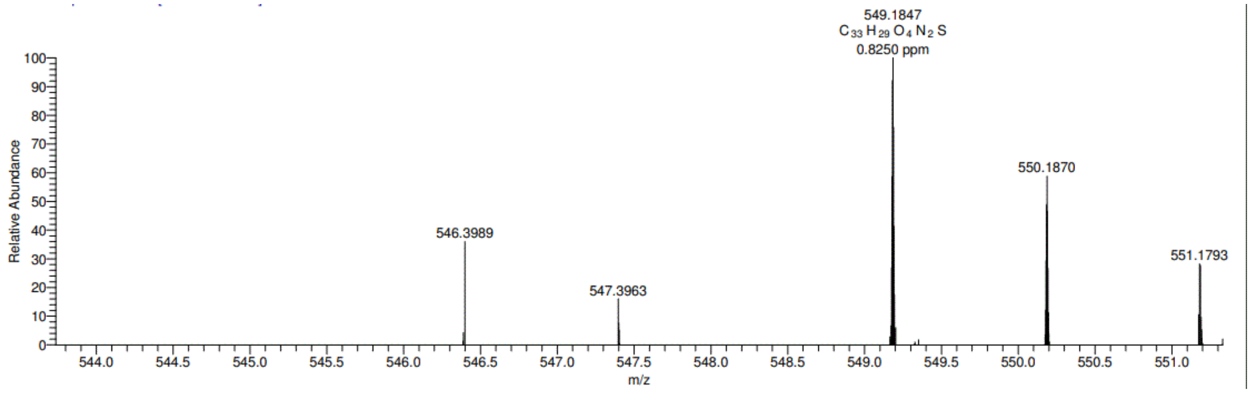

Figure S14. HRMS of **NPI-PTZ3**

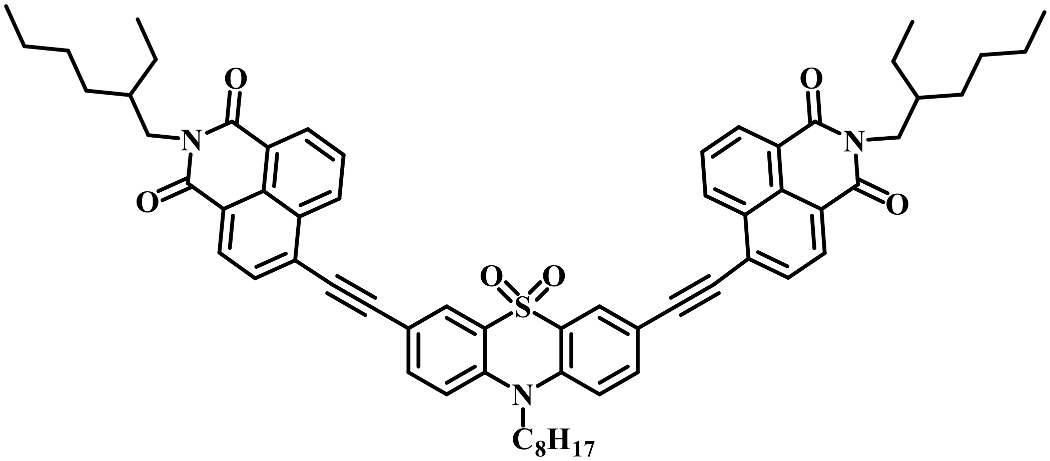

O
N
O
O
S
O
N
C_8H_{17}
O
N
O

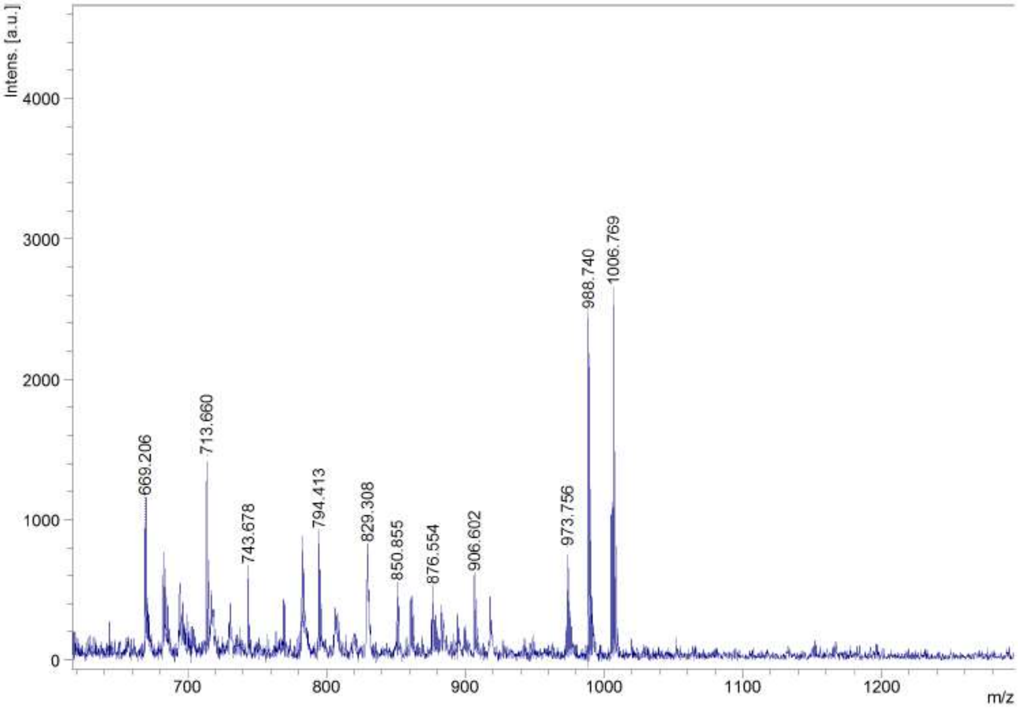

Intens. [a.u.]
4000
3000
2000
1000
0
669.206
713.660
743.678
794.413
829.308
850.855
876.554
906.602
973.756
988.740
1006.769
700
800
900
1000
1100
1200
m/z

Figure S15. MALDI spectra of **NPI-PTZ5**.

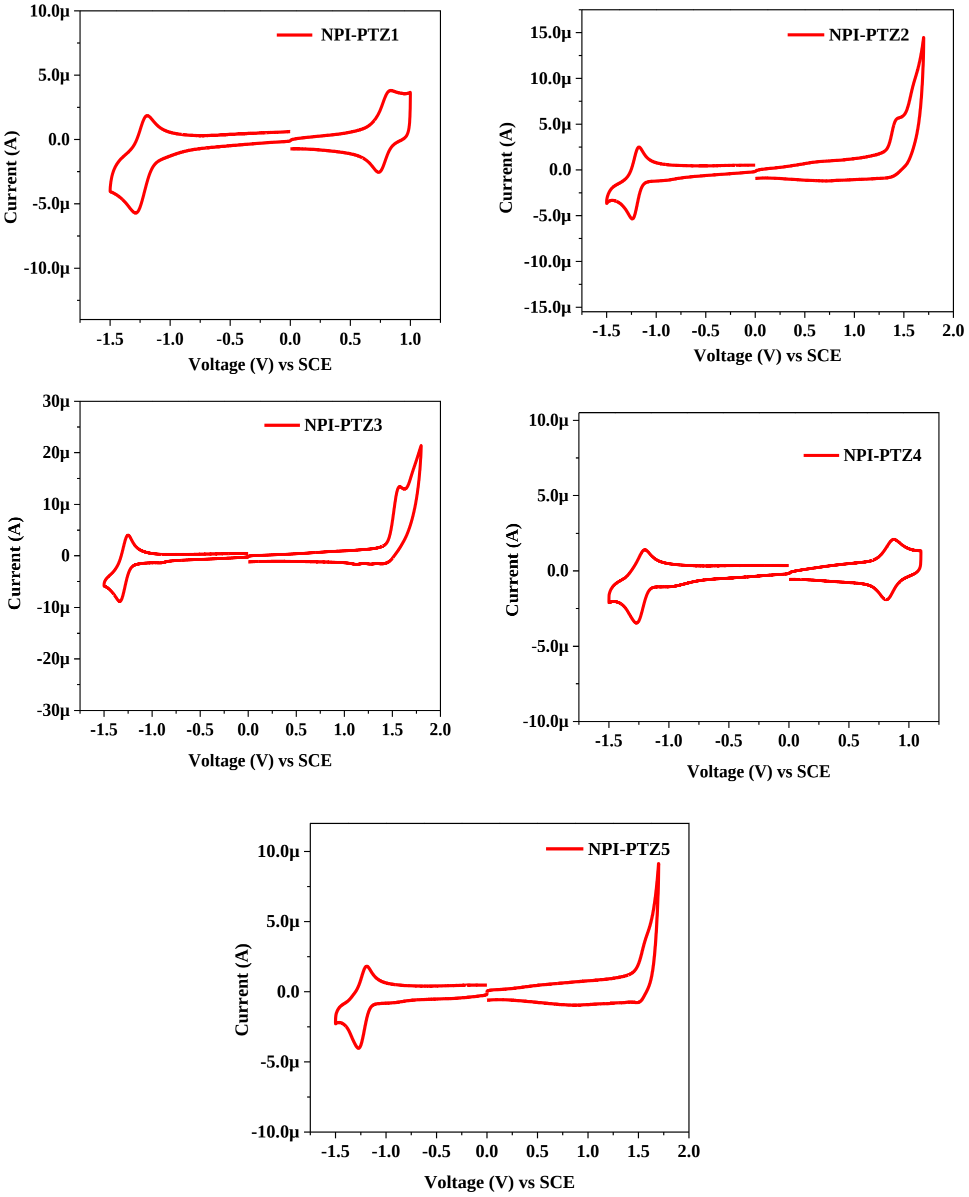

Figure S16. Cyclic voltammograms of **NPI-PTZ1–5** in 0.1 M solution of Bu_4NPF_6 in dichloromethane at 100 mV s^{-1} scan rate versus saturated calomel electrode (SCE) at 25 °C.

Table S1. Electrochemical data obtained by differential pulse voltammetry, in 0.1 M solution of Bu_4NPF_6 in DCM at 100 mV s^{-1} scan rate versus SCE electrode.

Compound	E_{red} / V	E_{ox} / V
**NPI-PTZ1**	-1.23	0.78
**NPI-PTZ2**	-1.20	1.41
**NPI-PTZ3**	-1.21	1.50
**NPI-PTZ4**	-1.23	0.83
**NPI-PTZ5**	-1.23	1.56

DFT calculations

NPI-PTZ1

Center Number	Atomic Number	Atomic Type	Coordinates (Angstroms) X	Y	Z
1	6	0	2.310566	3.961609	0.240309
2	6	0	1.243692	3.360270	-0.438778
3	6	0	3.521461	3.290422	0.401042
4	6	0	1.416563	2.053058	-0.925553
5	6	0	3.679508	1.996072	-0.079835
6	6	0	-1.192087	3.317920	-0.525906
7	6	0	2.614904	1.373947	-0.728774
8	6	0	-2.340969	3.879513	0.052212
9	6	0	-1.276275	1.996290	-1.011234
10	1	0	2.714454	0.360385	-1.099569
11	6	0	-3.528956	3.165981	0.126724
12	1	0	-2.309691	4.882424	0.455495
13	6	0	-2.450431	1.270027	-0.901057
14	6	0	-3.608918	1.846753	-0.345094
15	1	0	-2.484897	0.251480	-1.267272
16	7	0	0.012393	4.031552	-0.636689
17	16	0	0.114045	1.303922	-1.884160
18	6	0	-0.019680	5.477260	-0.463967
19	1	0	-0.887926	5.878340	-0.986711
20	1	0	0.873952	5.906661	-0.916124
21	1	0	4.335503	3.784072	0.919003
22	1	0	-4.402936	3.627653	0.569091
23	1	0	2.201214	4.956112	0.651455
24	6	0	-4.814502	1.103612	-0.264399
25	6	0	-5.836197	0.453364	-0.206422
26	6	0	-7.015343	-0.330552	-0.155757
27	6	0	-8.229389	0.192249	0.410336

28	6	0	-7.006409	-1.628946	-0.662901
29	6	0	-8.326101	1.497439	0.948153
30	6	0	-9.387961	-0.638789	0.431898
31	6	0	-8.153734	-2.429200	-0.632569
32	1	0	-6.090077	-2.018771	-1.087849
33	6	0	-9.510789	1.958516	1.480166
34	1	0	-7.447917	2.131354	0.934167
35	6	0	-10.594274	-0.142573	0.984057
36	6	0	-9.334913	-1.951067	-0.095538
37	1	0	-8.136654	-3.436591	-1.029358
38	6	0	-10.651832	1.136876	1.499462
39	1	0	-9.566864	2.961168	1.887430
40	1	0	-11.589323	1.485826	1.914300
41	6	0	-11.810135	-0.992889	1.015523
42	6	0	-10.530838	-2.826889	-0.081458
43	6	0	-12.908661	-3.111162	0.520945
44	1	0	-12.658642	-4.082784	0.107035
45	1	0	-13.254175	-3.207710	1.550410
46	8	0	-10.515192	-3.958918	-0.529334
47	8	0	-12.869802	-0.612483	1.478562
48	7	0	-11.697496	-2.282409	0.480675
49	1	0	4.616338	1.468372	0.052039
50	1	0	-0.066469	5.792224	0.587655
51	1	0	-13.700618	-2.639817	-0.062479

HF=-1736.3162779 Hartree

NPI-PTZ4

Center Number	Atomic Number	Atomic Type	Coordinates (Angstroms) X	Y	Z
1	6	0	2.533142	3.647037	-3.541563
2	6	0	1.471964	2.731632	-3.599184
3	6	0	3.701099	3.348781	-2.853937
4	6	0	1.617764	1.507796	-2.916829
5	6	0	3.843110	2.133122	-2.167013
6	6	0	-0.953099	2.587254	-3.799666
7	6	0	2.768244	1.224768	-2.198372
8	6	0	-2.110140	3.369877	-3.926055
9	6	0	-1.062872	1.348317	-3.138176
10	1	0	2.848056	0.283079	-1.669691
11	6	0	-3.331732	2.928281	-3.437512
12	1	0	-2.060811	4.338271	-4.404655
13	6	0	-2.273960	0.924069	-2.615611
14	6	0	-3.439561	1.697845	-2.770964
15	1	0	-2.327473	-0.026011	-2.098757
16	7	0	0.289227	3.008820	-4.311171
17	16	0	0.350845	0.265591	-3.079049
18	6	0	0.305729	4.090944	-5.288152

19	1	0	-0.513510	3.945547	-5.991676
20	1	0	1.240240	4.049009	-5.846785
21	1	0	4.509485	4.068778	-2.833312
22	1	0	-4.212044	3.547160	-3.557255
23	1	0	2.449180	4.607329	-4.031632
24	6	0	-4.685210	1.247763	-2.261925
25	6	0	-5.754439	0.869410	-1.834286
26	6	0	-7.016219	0.442086	-1.349966
27	6	0	-7.149000	-0.791893	-0.624106
28	6	0	-8.148336	1.221037	-1.581010
29	6	0	-6.051616	-1.639252	-0.340611
30	6	0	-8.442938	-1.180959	-0.168503
31	6	0	-9.409865	0.822952	-1.124142
32	1	0	-8.042782	2.149634	-2.127642
33	6	0	-6.225797	-2.813896	0.358291
34	1	0	-5.066708	-1.347066	-0.683608
35	6	0	-8.597089	-2.394206	0.545945
36	6	0	-9.567102	-0.360992	-0.427779
37	1	0	-10.284424	1.434006	-1.309219
38	6	0	-7.503582	-3.196174	0.804261
39	1	0	-5.373945	-3.450259	0.567038
40	1	0	-7.655479	-4.116872	1.353734
41	6	0	-9.936758	-2.817427	1.024506
42	6	0	-10.919870	-0.753222	0.037123
43	6	0	-12.330528	-2.401547	1.215838
44	1	0	-13.052924	-1.644313	0.928519
45	1	0	-12.586371	-3.363958	0.771437
46	8	0	-11.904016	-0.067903	-0.172133
47	8	0	-10.121352	-3.850926	1.640150
48	7	0	-11.012222	-1.967163	0.736817
49	6	0	5.031921	1.830066	-1.454136
50	6	0	6.052171	1.579812	-0.849376
51	6	0	7.254661	1.309544	-0.149248
52	6	0	7.444089	0.061691	0.539286
53	6	0	8.271818	2.261731	-0.124497
54	6	0	6.464233	-0.959006	0.564695
55	6	0	8.673333	-0.159480	1.227263
56	6	0	9.471458	2.026130	0.556744
57	1	0	8.124061	3.199787	-0.644571
58	6	0	6.689122	-2.140028	1.237971
59	1	0	5.529361	-0.795593	0.042794
60	6	0	8.881286	-1.381837	1.911984
61	6	0	9.680331	0.835086	1.227012
62	1	0	10.256411	2.771894	0.570485
63	6	0	7.902426	-2.355180	1.915827
64	1	0	5.927540	-2.910795	1.247044
65	6	0	10.154634	-1.630266	2.632974
66	6	0	10.964231	0.619891	1.937882
67	1	0	8.092907	-3.278250	2.449062
68	8	0	10.380500	-2.662843	3.236307
69	7	0	11.115256	-0.611355	2.595903
70	8	0	11.846550	1.458427	1.955715

71	6	0	12.369282	-0.873817	3.313198
72	1	0	13.017416	-0.014817	3.172191
73	1	0	12.164658	-1.028216	4.373321
74	1	0	0.209692	5.087480	-4.836970
75	1	0	12.835659	-1.777417	2.920113
76	1	0	-12.308879	-2.520461	2.299475

HF=-2517.5267828 Hartree

NPI-PTZ2

Center Number	Atomic Number	Atomic Type	Coordinates (Angstroms) X	Y	Z
1	6	0	2.255758	3.872602	0.311760
2	6	0	1.198646	3.374283	-0.461070
3	6	0	3.368765	3.077127	0.578119
4	6	0	1.317137	2.068039	-0.958528
5	6	0	3.445135	1.768113	0.110120
6	6	0	-1.211997	3.439320	-0.661393
7	6	0	2.406257	1.261355	-0.667023
8	6	0	-2.367093	3.993717	-0.087464
9	6	0	-1.310388	2.132212	-1.169501
10	1	0	2.421404	0.249535	-1.056053
11	6	0	-3.541349	3.258399	-0.007363
12	1	0	-2.345144	4.990674	0.331531
13	6	0	-2.461814	1.378823	-1.064175
14	6	0	-3.612086	1.933299	-0.472980
15	1	0	-2.462660	0.365634	-1.448630
16	7	0	0.018447	4.118512	-0.703603
17	16	0	0.076078	1.473036	-2.140145
18	6	0	0.034582	5.552385	-0.445297
19	1	0	-0.791191	6.019438	-0.981521
20	1	0	0.964386	5.970838	-0.829429
21	1	0	4.173706	3.485376	1.178463
22	1	0	-4.417494	3.706728	0.444762
23	1	0	2.204018	4.867041	0.734521
24	6	0	-4.806737	1.175380	-0.364555
25	6	0	-5.817342	0.511670	-0.280995
26	6	0	-6.982813	-0.291279	-0.202175
27	6	0	-8.191932	0.213636	0.389092
28	6	0	-6.962116	-1.590332	-0.706011
29	6	0	-8.298646	1.518566	0.925894
30	6	0	-9.335680	-0.636420	0.438178
31	6	0	-8.095349	-2.409658	-0.648358
32	1	0	-6.048677	-1.965925	-1.149534
33	6	0	-9.478897	1.961137	1.482627
34	1	0	-7.431790	2.167116	0.891476

35	6	0	-10.537789	-0.158935	1.015394
36	6	0	-9.271893	-1.949133	-0.086924
37	1	0	-8.069944	-3.417797	-1.042686
38	6	0	-10.605492	1.120645	1.528897
39	1	0	-9.542869	2.963740	1.888857
40	1	0	-11.539322	1.455057	1.963459
41	6	0	-11.738454	-1.029388	1.075529
42	6	0	-10.453065	-2.844999	-0.044156
43	6	0	-12.811970	-3.167302	0.610941
44	1	0	-12.554668	-4.136095	0.194883
45	1	0	-13.133499	-3.265942	1.647924
46	8	0	-10.427838	-3.977475	-0.489662
47	8	0	-12.793613	-0.665116	1.560983
48	7	0	-11.615976	-2.318448	0.541908
49	1	0	4.305183	1.150912	0.339400
50	1	0	-0.050369	5.802418	0.620713
51	1	0	-13.624025	-2.711346	0.043158
52	8	0	0.034394	-0.033569	-2.101550

HF=-1811.5059713

NPI-PTZ3

Center Number	Atomic Number	Atomic Type	Coordinates (Angstroms) X	Y	Z
1	6	0	2.356562	3.955441	0.176535
2	6	0	1.245245	3.371613	-0.454983
3	6	0	3.544557	3.247593	0.313989
4	6	0	1.399125	2.060173	-0.940820
5	6	0	3.664289	1.937011	-0.148013
6	6	0	-1.196724	3.355020	-0.539151
7	6	0	2.579753	1.341813	-0.777594
8	6	0	-2.368927	3.919782	-0.000958
9	6	0	-1.293287	2.035063	-1.023632
10	1	0	2.629417	0.328315	-1.156952
11	6	0	-3.545385	3.193773	0.061121
12	1	0	-2.354740	4.922065	0.403856
13	6	0	-2.460072	1.293007	-0.939833
14	6	0	-3.618821	1.861761	-0.391981
15	1	0	-2.465963	0.277043	-1.314424
16	7	0	0.016346	4.048267	-0.549873
17	16	0	0.090652	1.377192	-1.929924
18	6	0	-0.000294	5.498144	-0.369640
19	1	0	-0.863679	5.911399	-0.888805
20	1	0	0.893416	5.922009	-0.824697
21	1	0	4.382202	3.723429	0.810900
22	1	0	-4.427358	3.653105	0.490303
23	1	0	2.287608	4.951698	0.590732
24	6	0	-4.822980	1.116535	-0.303833

25	6	0	-5.842907	0.465736	-0.237753
26	6	0	-7.019358	-0.323427	-0.177978
27	6	0	-8.232765	0.197680	0.389323
28	6	0	-7.003527	-1.624061	-0.677074
29	6	0	-8.335142	1.506064	0.918662
30	6	0	-9.386263	-0.639890	0.421257
31	6	0	-8.146635	-2.430957	-0.636782
32	1	0	-6.086771	-2.011148	-1.103532
33	6	0	-9.520381	1.963308	1.452200
34	1	0	-7.461324	2.145737	0.896879
35	6	0	-10.593282	-0.147465	0.975080
36	6	0	-9.327302	-1.955255	-0.097665
37	1	0	-8.125422	-3.440653	-1.027277
38	6	0	-10.656539	1.134860	1.481959
39	1	0	-9.581205	2.968406	1.852668
40	1	0	-11.594319	1.481128	1.898329
41	6	0	-11.804139	-1.005093	1.017533
42	6	0	-10.519085	-2.838631	-0.072159
43	6	0	-12.892441	-3.133338	0.542082
44	1	0	-12.638383	-4.106495	0.134336
45	1	0	-13.233126	-3.224315	1.573610
46	8	0	-10.497162	-3.972988	-0.512481
47	8	0	-12.863393	-0.627435	1.482963
48	7	0	-11.686215	-2.297526	0.491001
49	1	0	4.590398	1.390425	-0.021895
50	1	0	-0.040128	5.797387	0.684655
51	1	0	-13.689235	-2.670887	-0.041779
52	8	0	0.100195	-0.084489	-1.838025
53	8	0	0.119349	2.020293	-3.246724

HF=-1886.7347916

NPI-PTZ5

Center Number	Atomic Number	Atomic Type	Coordinates (Angstroms) X	Y	Z
1	6	0	2.581114	3.627908	-3.704419
2	6	0	1.485854	2.744987	-3.742547
3	6	0	3.725726	3.312100	-2.993419
4	6	0	1.625709	1.529643	-3.044249
5	6	0	3.838772	2.109463	-2.267840
6	6	0	-0.942644	2.599736	-3.947980
7	6	0	2.758080	1.217783	-2.308915
8	6	0	-2.125465	3.346858	-4.100650
9	6	0	-1.052243	1.368602	-3.272549
10	1	0	2.796206	0.274768	-1.778265
11	6	0	-3.327308	2.889997	-3.588527

12	1	0	-2.102929	4.309529	-4.591843
13	6	0	-2.246674	0.916555	-2.734826
14	6	0	-3.416854	1.673910	-2.882357
15	1	0	-2.260568	-0.029711	-2.208802
16	7	0	0.293733	3.081004	-4.395846
17	16	0	0.364992	0.292620	-3.260424
18	6	0	0.312468	4.164134	-5.378385
19	1	0	-0.504373	4.018548	-6.083269
20	1	0	1.244630	4.118497	-5.938984
21	1	0	4.547956	4.016622	-2.973481
22	1	0	-4.218634	3.492660	-3.711567
23	1	0	2.528347	4.584881	-4.204461
24	6	0	-4.649838	1.232651	-2.336197
25	6	0	-5.703778	0.863416	-1.866589
26	6	0	-6.942104	0.446883	-1.314952
27	6	0	-7.069519	-0.834933	-0.677600
28	6	0	-8.050331	1.287689	-1.385880
29	6	0	-5.995796	-1.750443	-0.566758
30	6	0	-8.335126	-1.202598	-0.133417
31	6	0	-9.284821	0.908572	-0.845424
32	1	0	-7.947767	2.251903	-1.867633
33	6	0	-6.166506	-2.969136	0.052759
34	1	0	-5.032249	-1.478040	-0.979417
35	6	0	-8.485485	-2.460712	0.499508
36	6	0	-9.435393	-0.317031	-0.224490
37	1	0	-10.141496	1.568230	-0.903414
38	6	0	-7.415977	-3.328707	0.589373
39	1	0	-5.333496	-3.657889	0.128129
40	1	0	-7.563808	-4.283342	1.078733
41	6	0	-9.795152	-2.860030	1.072668
42	6	0	-10.758557	-0.684271	0.339254
43	6	0	-12.135297	-2.352338	1.529283
44	1	0	-12.843389	-1.546715	1.363774
45	1	0	-12.478110	-3.269282	1.049028
46	8	0	-11.720158	0.059276	0.275505
47	8	0	-9.975015	-3.929035	1.625140
48	7	0	-10.847446	-1.941847	0.955368
49	6	0	5.008442	1.811697	-1.521919
50	6	0	6.009761	1.565311	-0.886024
51	6	0	7.189245	1.293555	-0.147008
52	6	0	7.343448	0.055093	0.565681
53	6	0	8.214506	2.235743	-0.109291
54	6	0	6.351704	-0.954677	0.579575
55	6	0	8.550769	-0.167766	1.290893
56	6	0	9.392083	1.998849	0.609597
57	1	0	8.091840	3.166527	-0.648628
58	6	0	6.545654	-2.126818	1.277186
59	1	0	5.432951	-0.791879	0.029729
60	6	0	8.726985	-1.381259	1.999685
61	6	0	9.567853	0.816418	1.303372
62	1	0	10.184572	2.736245	0.634099
63	6	0	7.737833	-2.343962	1.991368

64	1	0	5.775716	-2.889143	1.276801
65	6	0	9.977726	-1.631641	2.758725
66	6	0	10.830021	0.599088	2.053813
67	1	0	7.903324	-3.260654	2.543568
68	8	0	10.176400	-2.656900	3.383021
69	7	0	10.950150	-0.622865	2.733125
70	8	0	11.718782	1.430211	2.083234
71	6	0	12.181329	-0.887761	3.488471
72	1	0	12.840830	-0.036038	3.356395
73	1	0	11.945272	-1.028591	4.543859
74	1	0	0.218106	5.155678	-4.920482
75	1	0	12.650633	-1.799554	3.118528
76	1	0	-12.018504	-2.547832	2.595631
77	8	0	0.317377	-0.586403	-2.090273
78	8	0	0.514206	-0.286401	-4.597810

HF=-2667.9443694 Hartree

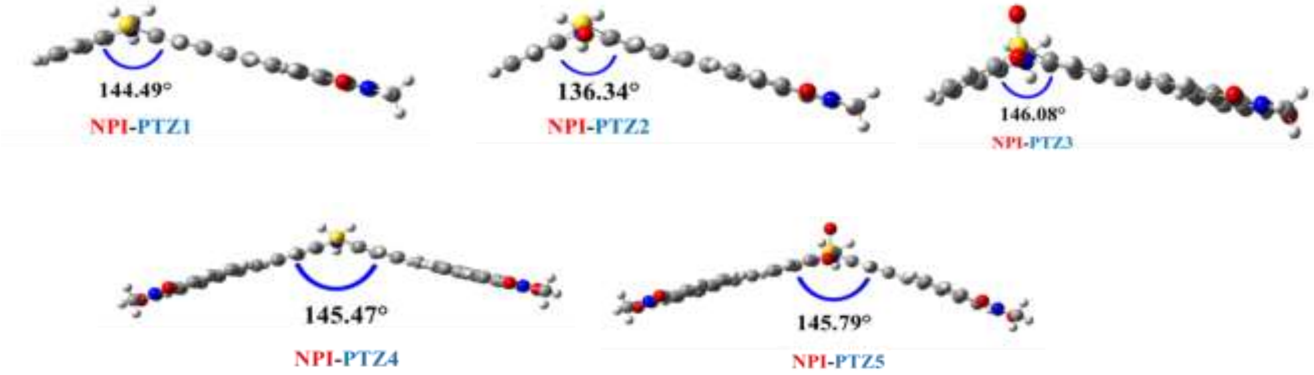

Figure S17. Lateral view of the ground state optimized geometries and dihedral angles for the investigated compounds.

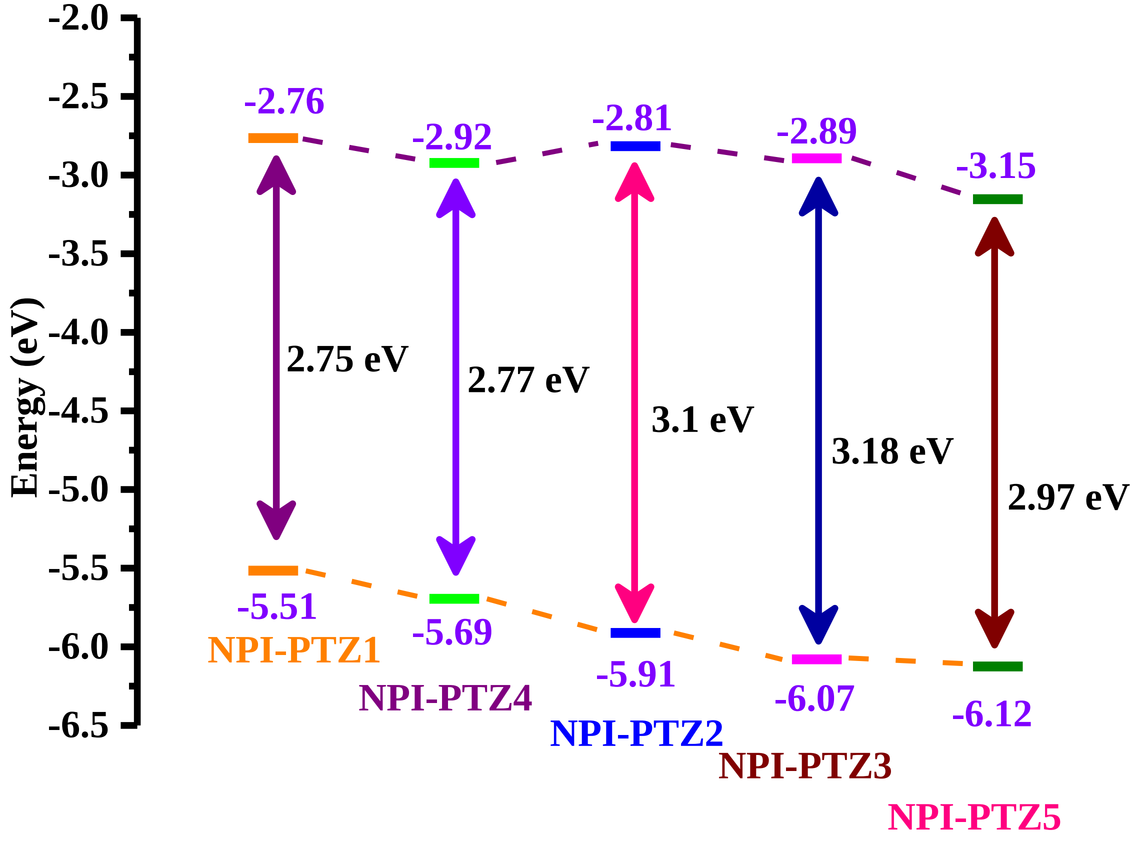

Figure S18. Energy levels diagram of the frontier orbitals estimated by DFT calculations.

Table S2. Calculated electronic absorption transitions for the investigated molecules in dichloromethane by using CAM-B3LYP/6-31G** on the optimized structures.

**compound**	**λ_{calc} / nm**	**Composition**	**f [a]**	**λ_{exp}/ nm**
**NPI-PTZ1**	396	HOMO→LUMO (0.58)	1.22	440
**NPI-PTZ4**	408	HOMO→LUMO (0.56)	1.78	441
**NPI-PTZ2**	382	HOMO→LUMO (0.61)	1.30	415
**NPI-PTZ3**	367	HOMO→LUMO (0.63)	1.40	403
**NPI-PTZ5**	388	HOMO→LUMO (0.55)	2.09	423

[a] f oscillator strength

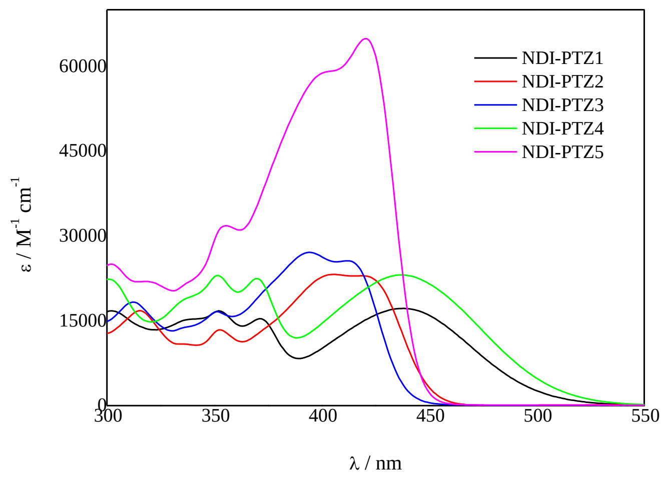

Figure S19. Molar extinction coefficients of the investigated compounds in Tol.

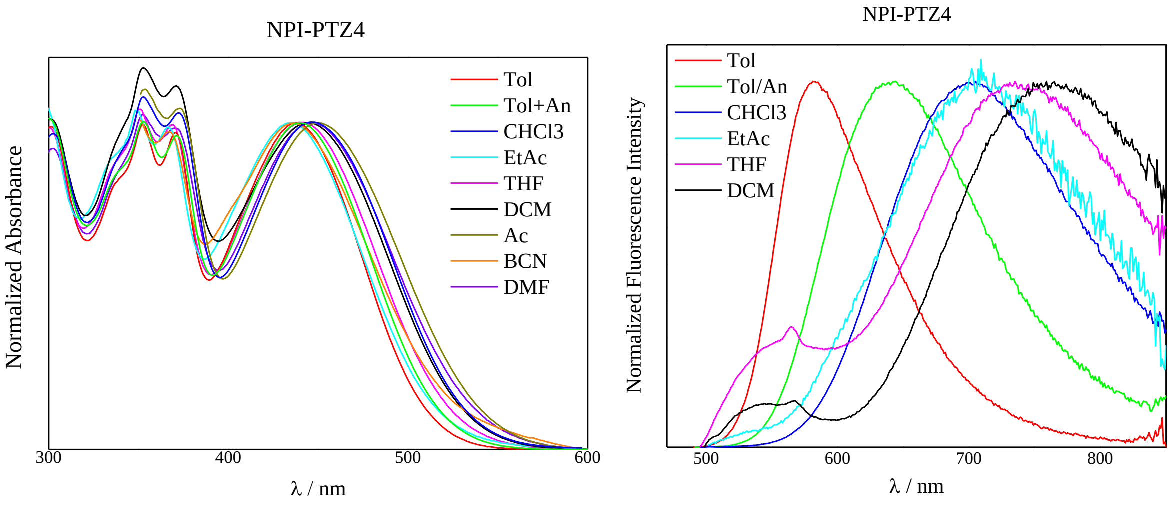

Figure S20. Solvent effect on the absorption (left) and emission (right) spectra of **NPI-PTZ4**.

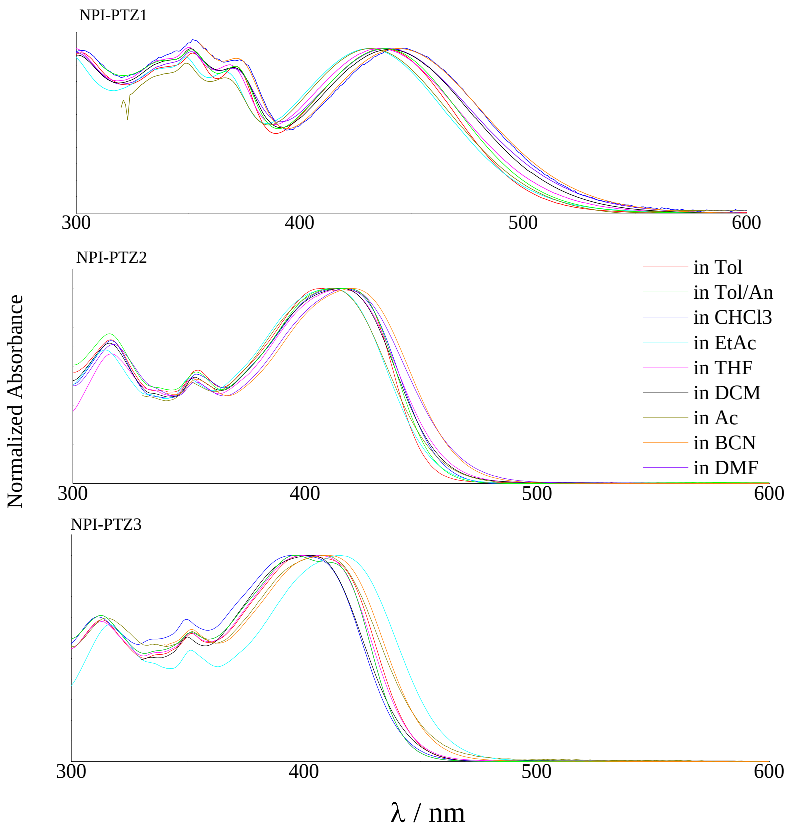

Figure S21. Solvent effect on the absorption spectra of the dipolar compounds.

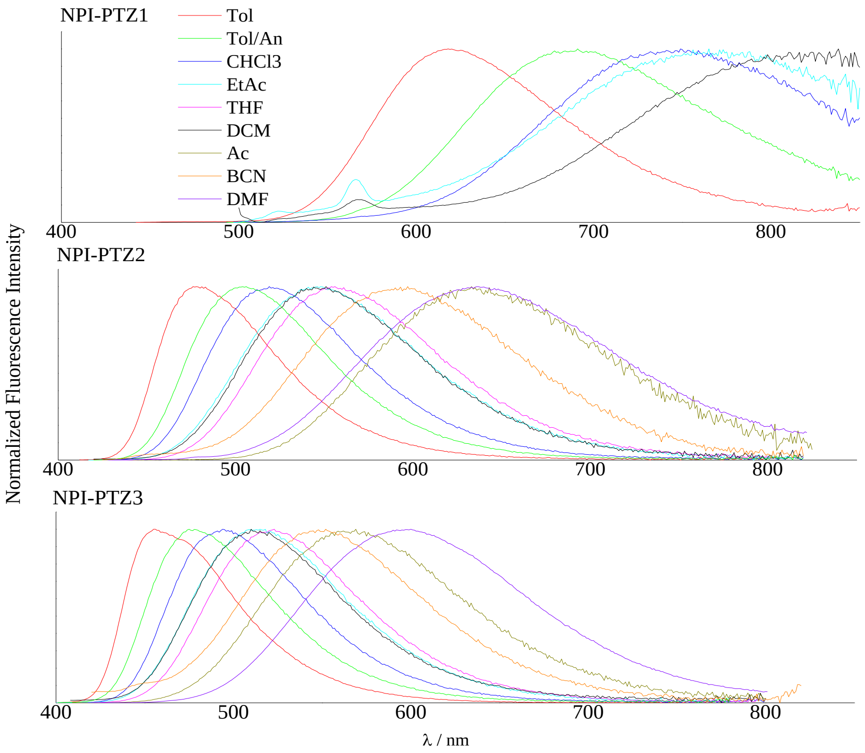

Figure S22. Solvent effect on the emission spectra of the dipolar compounds.

Table S3. Spectral properties of the investigated compounds.

**Compound**	**Solvent**	***f*** **(ε, n²)**	**λabs / nm**	**λem / nm**	**Δν / cm⁻¹**
NPI-PTZ1	Tol	0.0242	437	618	6702
	Tol/An 50:50	0.143	437	690	8391
	CHCl3	0.293	446	746	9017
	EtAc	0.4	432	765	10076
	THF	0.441	436	783	10164
	DCM	0.474	440	827	10635
	Ac	0.651	432		
	BCN	0.586	445		
	DMF	0.664	439		
NPI-PTZ 2	Tol	0.0242	407	478	3650
	Tol/An 50:50	0.143	415	504	4255
	CHCl3	0.293	416	519	4771
	EtAc	0.4	412	548	6024
	THF	0.441	415	554	6046

	DCM	0.474	415	545	5748
	Ac	0.651	412	610	7878
	BCN	0.586	420	593	6946
	DMF	0.664	418	637	8225
NPI-PTZ3	Tol	0.0242	395	456	3387
	Tol/An 50:50	0.143	396	478	4332
	CHCl3	0.293	402	494	4633
	EtAc	0.4	395	513	5823
	THF	0.441	404	523	5632
	DCM	0.474	403	512	5283
	Ac	0.651	404	562	6959
	BCN	0.586	415	548	5848
	DMF	0.664	408	597	7759
NPI-PTZ4	Tol	0.0242	438	582	5649
	Tol/An 50:50	0.143	440	642	7151
	CHCl3	0.293	448	702	8076
	EtAc	0.4	434	709	8937
	THF	0.441	441	735	9070
	DCM	0.474	445	765	9400
	Ac	0.651	436		
	BCN	0.586	450		
	DMF	0.664	438		
NPI-PTZ5	Tol	0.0242	421	448	1432
	Tol/An 50:50	0.143	422	462	2052
	CHCl3	0.293	423	481	2851
	EtAc	0.4	418	501	3963
	THF	0.441	421	508	4068
	DCM	0.474	423	503	3760
	Ac	0.651	419	559	5977
	BCN	0.586	426	545	5126
	DMF	0.664	424	596	6806

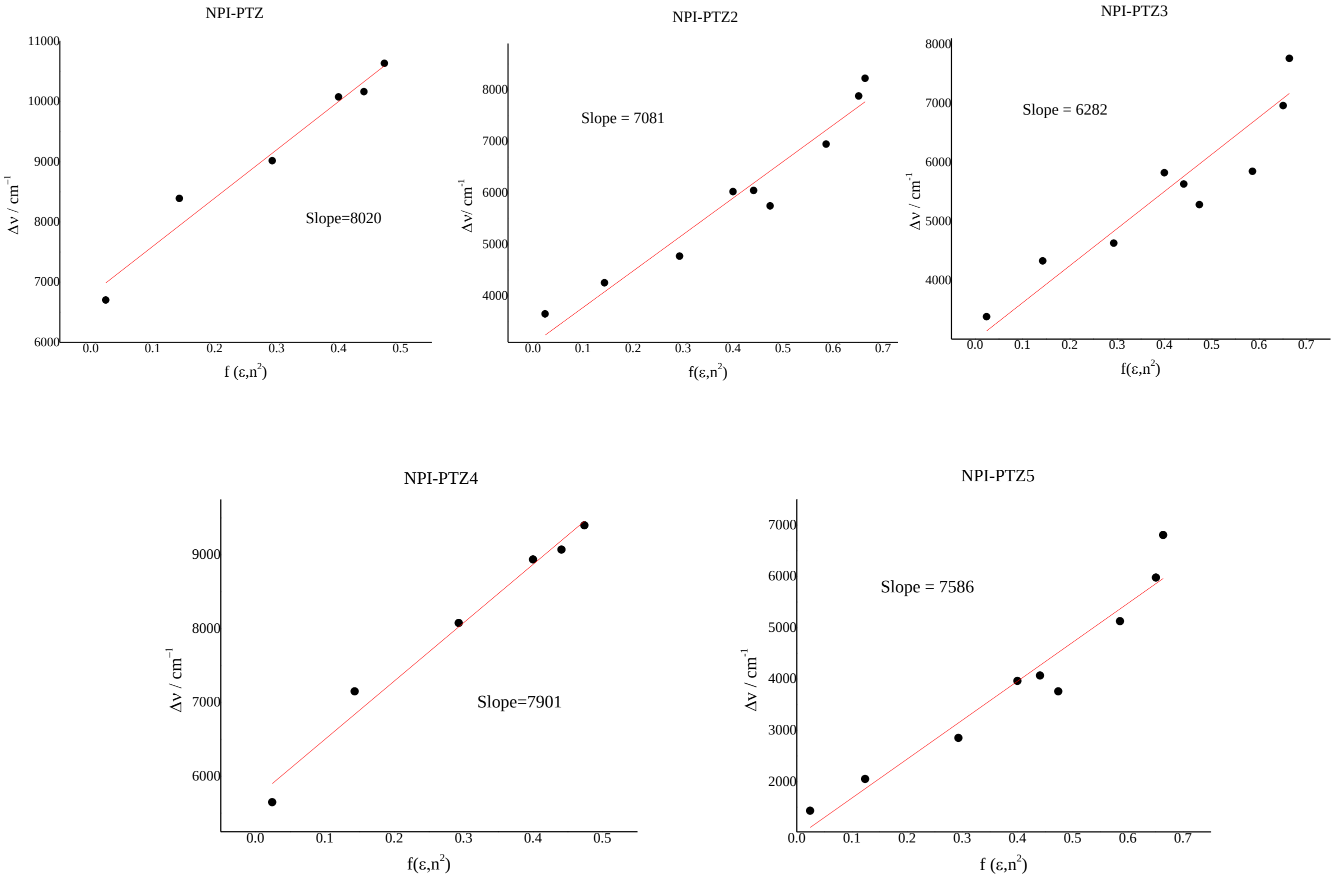

Figure S23. Plot of the Stokes shift as a function of the solvent properties for the investigated compounds.

Table S4. Calculated parameters (Onsager cavity radius, a, and distance between the two dipoles in the quadrupolar systems, d) and experimental parameters (frequency of the absorption maximum in a non-polar solvent, ν_{eg}, oscillator strength, f, difference of dipole moment between excited and ground state, Δμ, and hyperpolarizability coefficient, β_{CT} and β_0) derived by the fluorosolvatochromism of the investigated compounds using eqs. 4 and 6.

**Compound**	**a / Å**	**d / Å**	**Slope**	**Δμ / D**	**ν_{eg} /cm^{-1}**	***f***	**β_{CT} /10^{-30} esu^{-1} cm^5**	**β_0 /10^{-30} esu^{-1} cm^5**
**NPI-PTZ1**	10.29		8020	29.4	22883	0.303	224	60.7
**NPI-PTZ2**	10.10		7081	26.9	24570	0.401	167	59.1
**NPI-PTZ3**	10.30		6282	26.1	25316	0.455	154	59.5
**NPI-PTZ4**	13.35	11.87	7901	24.3	22831	0.433	269	71.8
**NPI-PTZ5**	13.15	11.78	7586	23.1	23753	1.043	463	146

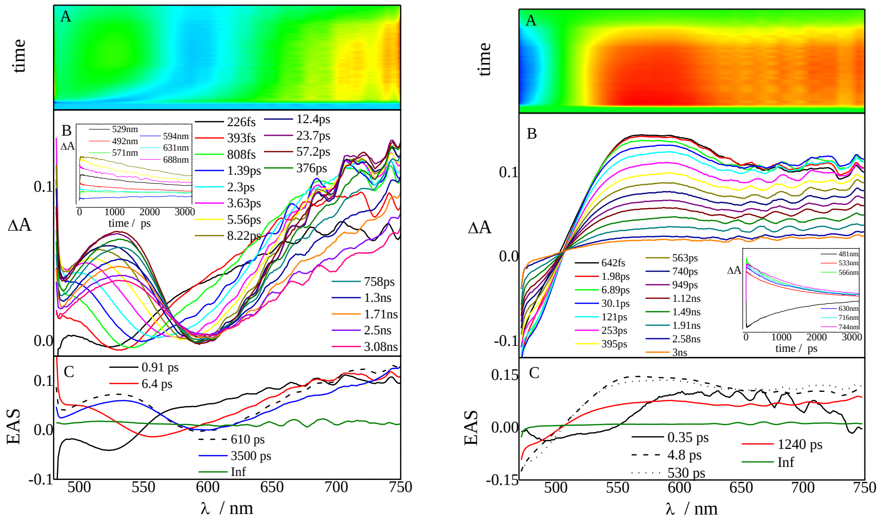

Figure S24. Femtosecond transient absorption spectroscopy of **NPI-PTZ4** (left) and **NPI-PTZ5** (right) in Tol.

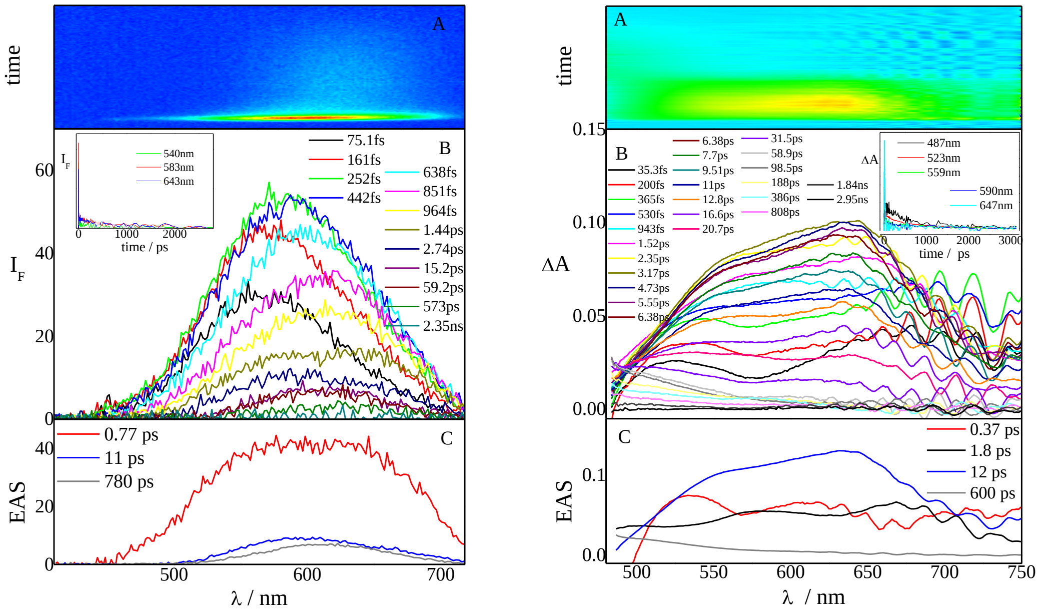

Figure S25. Femtosecond fluorescence up conversion (left) and transient absorption (right) spectroscopy of **NPI-PTZ4** in DMF.

Table S5. Results of the global fitting of the femtosecond transient absorption (TA) and fluorescence up conversion (FUC) data.

Compound	Solvent	τ_{TA} /ps	τ_{FUC} /ps	Assignment
NPI-PTZ1	Tol	1.4	1.7	Solv.
		6.0	5.2	S_1 (LE)
		199	191	SR
		5170	5170	S_1 (ICT)
		Inf		T_1
	DMF	0.15	0.18	Solv./S_1(LE)
		1.9	0.43	Solv.
		5.5	5.0	S_1(ICT)
		300	341	conformer
NPI-PTZ2	Tol	5.9	6.9	Solv.
		113	170	SR
		2220	2220	S_1 (LE)
		Inf		T_1
	DMF	0.29	0.54	Solv.
		1.7	2.3	Solv./S_1(LE)
		256	86	SR
		1300	1300	S_1(ICT)
NPI-PTZ3	Tol	5.6	3.1	Solv.
		134	196	SR
		1690	1690	S_1 (LE)
		Inf		T_1
	DMF	0.34	1.1	Solv.
		2.7	3.6	Solv./S_1(LE)
		108	246	SR
		3770	3770	S_1(ICT)
NPI-PTZ4	Tol	0.91	2.2	Solv.
		6.4	8.7	S_1 (LE)
		610	650	SR
		3500	3500	S_1 (ICT)
		Inf		T_1
	DMF	0.37	0.77	Solv./S_1(LE)
		1.8		Solv.
		12	11	S_1(ICT)
		600	780	conformer
NPI-PTZ5	Tol	0.35		Solv.
		4.8	3.7	Solv.
		530	535	SR
		1240	1240	S_1 (LE)
		Inf		T_1
	DMF	0.67	1.2	Solv.
		3.6	4.8	Solv./S_1(LE)
		450	459	SR
		2380	2380	S_1(ICT)

Table S6. Results of the global fitting of the femtosecond transient absorption data of **NPI-PTZ4** and **NPI-PTZ5** in several solvents of increasing polarity.

Solvent	τ / ps<br>**NPI-PTZ4**	Assignment	τ / ps<br>**NPI-PTZ5**	Assignment
Tol	0.91<br>6.4<br>610<br>3500<br>Inf	Solv.$_i$<br>Solv.$_d$/S_1(LE)<br>SR<br>S_1(ICT)<br>T_1	0.35<br>4.8<br>530<br>1240<br>Inf	Solv.$_i$<br>Solv.$_d$<br>SR<br>S_1(LE)<br>T_1
Tol/An 50:50	2.8<br>9.1<br>2100<br>Inf	Solv.i/S_1(LE)<br>Solv.$_d$<br>S_1(ICT)<br>T_1	0.26<br>9.9<br>1200<br>Inf	Solv.$_i$<br>Solv.$_d$/S_1(LE)<br>S_1(ICT)<br>T_1
$CHCl_3$	0.34<br>8.4<br><br>1650<br>Inf	Solv.i<br>Solv.$_d$/S_1(LE)<br><br>S_1(ICT)<br>T_1	1.8<br>7.4<br>450<br>3200<br>Inf	Solv.$_d$<br>S_1(LE)<br>SR<br>S_1(ICT)<br>T_1
THF	1.3<br>5.5<br>260<br>1400	Solv.i/S_1(LE)<br>Solv.$_d$<br>S_1(ICT)<br>conformer	0.53<br>2.3<br>42<br>2700	Solv.$_i$<br>Solv.$_d$/S_1(LE)<br>SR<br>S_1(ICT)
DCM	1.3<br>208<br>2430	Solv.$_i$/S_1(LE)<br>S_1(ICT)<br>conformer		
Ac			0.54<br>1.4<br>240<br>3300	Solv.$_i$ /S_1(LE)<br>Solv.$_d$<br>SR<br>S_1(ICT)
DMF	0.37<br>1.8<br>12<br>600	Solv.i/S_1(LE)<br>Solv.$_d$<br>S_1(ICT)<br>conformer	0.67<br>3.6<br>450<br>2380	Solv.$_i$ /S_1(LE)<br>Solv.$_d$<br>SR<br>S_1(ICT)

Table S7. Charge transfer rate constant (k_{et}) and charge transfer free energy variation (ΔG_0), evaluated for **NPI-PTZ4** and **NPI-PTZ5** from the experimental data.

Compound	Solvent	τ_{LE} / ps	k_{et} / s^{-1}	$\Delta\upsilon$ / cm^{-1}	ΔG_0 / eV
**NPI-PTZ4**	Tol<br>Tol/An 50:50<br>THF<br>DCM	6.4<br>2.8<br>1.3<br>1.3	1.6×10^{11}<br>3.6×10^{11}<br>7.7×10^{11}<br>7.7×10^{11}	5649<br>7151<br>9070<br>9400	-0.700<br>-0.887<br>-1.12<br>-1.16
**NPI-PTZ5**	Tol<br>Tol/An 50:50<br>$CHCl_3$<br>THF<br>Ac<br>DMF	1240<br>9.9<br>7.4<br>2.3<br>0.54<br>0.67	8.1×10^{8}<br>1.0×10^{11}<br>1.4×10^{11}<br>4.4×10^{11}<br>1.8×10^{12}<br>1.5×10^{12}	1432<br>2052<br>2851<br>4068<br>5977<br>6806	-0.178<br>-0.254<br>-0.353<br>-0.504<br>-0.741<br>-0.844

k_{et} is obtained as $1/\tau_{LE}$; ΔG_0 is obtained as $-\Delta\upsilon$, where $\Delta\upsilon$ is the Stokes shift.

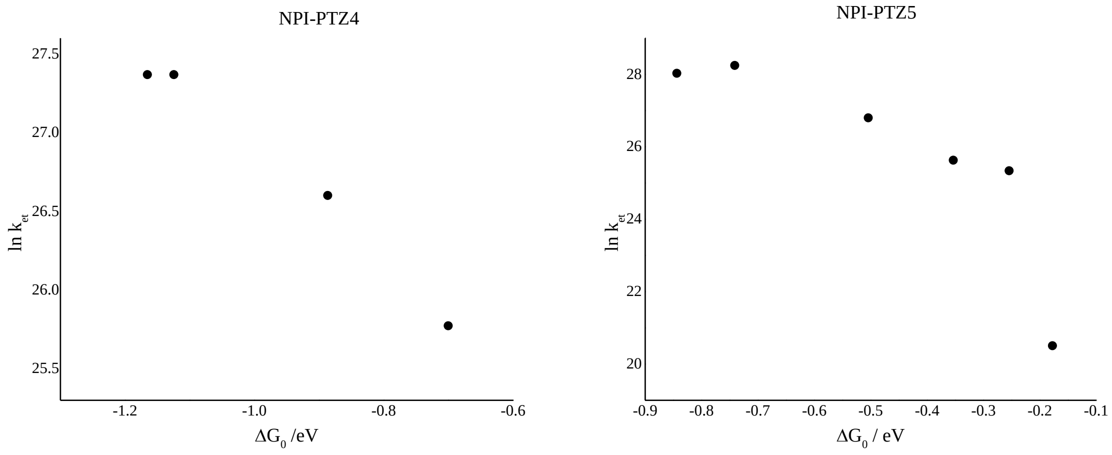

Figure S26. Dependence of the charge transfer rate constant (k_{et}) upon the charge transfer free energy variation (ΔG_0) in the context of the Marcus theory (detailed data in Table S7).

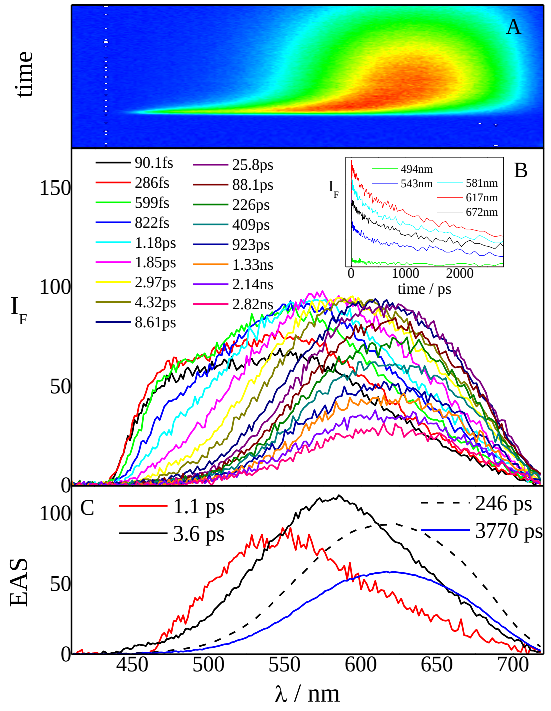

Figure S27. Femtosecond fluorescence up conversion spectroscopy of **NPI-PTZ3** in DMF.

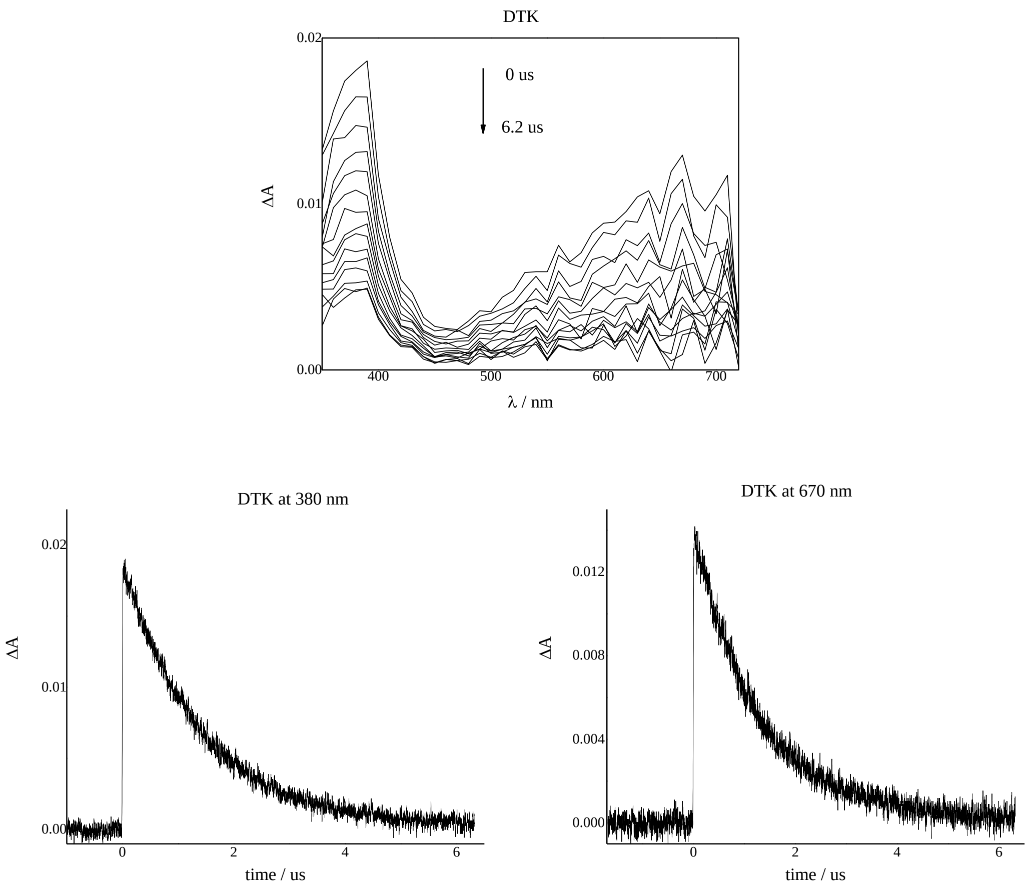

Figure S28. Triplet absorption spectra (upper graph) and decay kinetics (lower graphs) obtained for DTK in MeCN by nanosecond transient absorption.

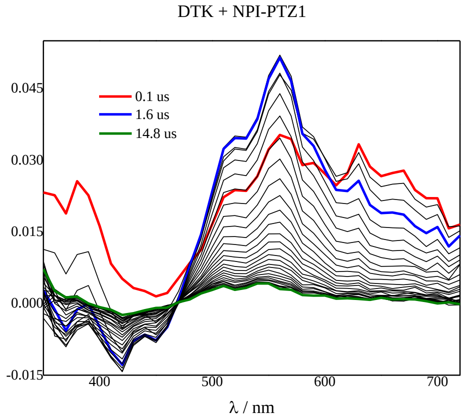

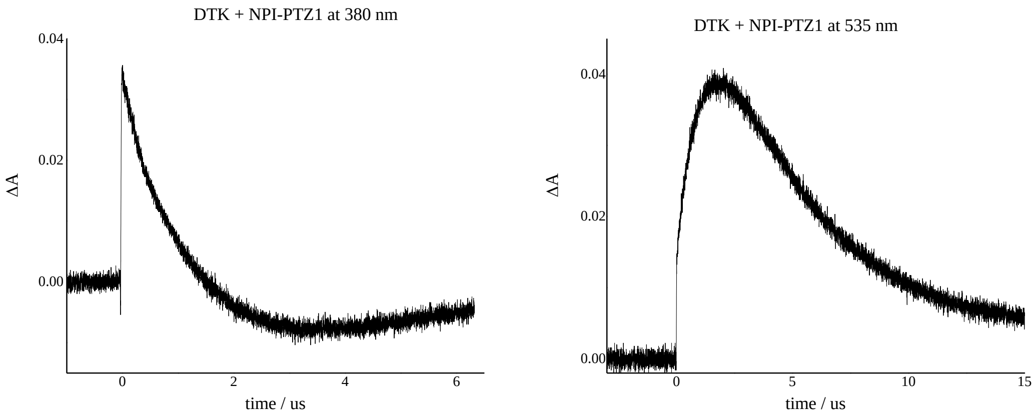

Figure S29. Triplet absorption spectra (upper graph) and decay kinetics (lower graphs) obtained during the sensitization experiment carried out by employing DTK as triplet energy donor and **NPI-PTZ1** as triplet energy acceptor in MeCN through nanosecond transient absorption.

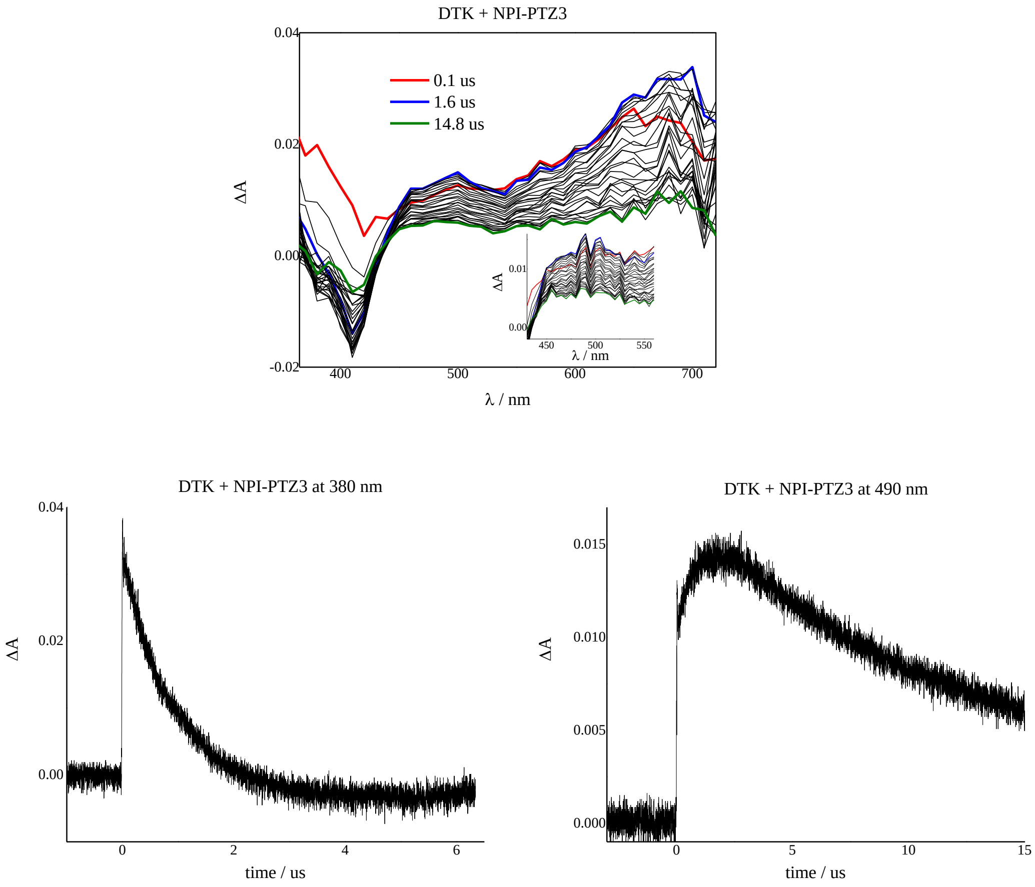

Figure S30. Triplet absorption spectra (upper graph) and decay kinetics (lower graphs) obtained during the sensitization experiment carried out by employing DTK as triplet energy donor and **NPI-PTZ3** as triplet energy acceptor in MeCN through nanosecond transient absorption.

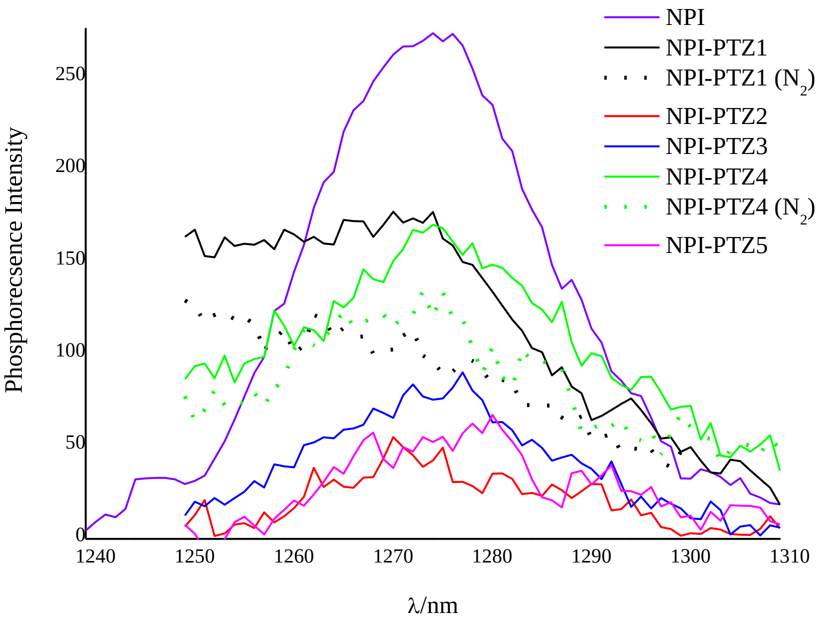

Figure S31. Phosphorescence spectra of the singlet oxygen produced by photoexcitation of the investigated compounds in air equilibrated Tol. For **NPI-PTZ1** and **NPI-PTZ4**, the spectra are distorted by the fluorescence signal interpherence, significantly red shifted for these samples and identified through the emission spectra recorded for the corresponding nitrogen purged solutions (dotted lines).
